# Study on the structural phase transitions in NaSICON-type compounds using Ag_3_Sc_2_(PO_4_)_3_ as a model system

**DOI:** 10.1107/S2052520620014870

**Published:** 2020-12-24

**Authors:** Günther J. Redhammer, Gerold Tippelt, Quirin Stahl, Artur Benisek, Daniel Rettenwander

**Affiliations:** a Department of Chemistry and Physics of Materials, Materials Science and Mineralogy Group, University of Salzburg, Jakob-Haringerstrasse 2A, Salzburg 5020, Austria; b Institut für Festkörper- und Materialphysik, Technische Universität Dresden, Haeckelstrasse 3, Dresden 01069, Germany; c Institute for Chemistry and Technology of Materials, Graz University of Technology, Stremayrgasse 9, Graz 8010, Austria

**Keywords:** NaSICON-type com­pound, Ag_3_Sc_2_(PO_4_)_3_, structural phase transition, order-disorder, ion conductivity, scandium

## Abstract

NaSICON (Na Super-Ionic CONducting) structured materials are among the most promising solid electrolytes for Li-ion batteries and ‘beyond Li-ion’ batteries (*e.g.* Na and K) due to their superior ionic conductivities. Although this material has been well known for decades, its exact phase behaviour is still poorly understood. Herein, a starting material of Na_3_Sc_2_(PO_4_)_3_ single crystals is used, grown by flux methodology, where Na is subsequently chemically replaced by Ag, in order to take advantage of the higher scattering contrast of Ag. It is found that the NaSICON-type com­pound shows two phase transitions from a low-tem­per­ature monoclinic α-phase to a monoclinic β-phase at about 180 K and to a rhombohedral γ-phase at about 290 K. The framework of [Sc_2_(PO_4_)_3_]^3−^ is rigid and does not change significantly with tem­per­ature and change of symmetry. The main driving force for the phase transitions is related to order–disorder phenomena of the conducting cations. The sensitivity of the phase behaviour on the ordering of these ions suggests that small com­positional changes can have a great impact on the phase behaviour and, hence, on the ionic conductivity of NaSICON-structured materials.

## Abstract 2

The superionic conducting Na Super-Ionic CONducting-type (NaSICON-type) material Ag_3_Sc_2_(PO_4_)_3_ was studied by single-crystal X-ray diffraction between 100 and 520 K. Two phase transitions were found, at 177 and 291 K. Full structural characterization of the three polymorphs is given for the first time.

## 1. Introduction   

NaSICON (Na Super-Ionic CONducting)-type com­pounds, with the general formula *A* *_x_MM*′(*T*O_4_)_3_ (Masquelier & Croguennec, 2013 ▸), are promising materials for Li-ion and ‘beyond Li-ion’ batteries. They are built from a framework of corner-sharing tetrahedra (*T*-sites) and octahedra (*M*-sites), with well-connected large interstitial voids that can host alkali-metal ions (Goodenough *et al.*, 1976 ▸). This three-dimensional (3D) alkali-metal ion sublattice enables a fast-ionic transport when used as a solid electrolyte and enables the insertion/extraction of ions during charge and discharge when used as an electrode material.

Due to the high potential for these materials to be used in battery applications, it is important to understand their structural properties so that a fundamental understanding of the origin of their superior properties can be gained. Generally, the mobility and site preference of alkali ions, as well as their dependence on tem­per­ature, are strongly connected to their phase behaviour. The symmetry of the high-tem­per­ature γ-polymorph (space group *R* 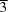 *c*) of NaSICON-type materials, *e.g.* *A*_3_ *M*_2_(PO_4_)_3_-type com­pounds (*M* = Sc, Cr, Fe and V), undergoes a reduction towards an orthorhombic, monoclinic or triclinic symmetry with decreasing tem­per­ature. For example, a phase transition sequence is observed in Na_3_V_2_(PO_4_)_3_ from an ordered monoclinic α-form to the classical disordered rhombohedral *R* 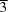 *c* structure with two intermediate incommensurately modulated phases β and β′, whose structures are still not resolved (Chotard *et al.*, 2015 ▸). Similar phase-transition sequences from a low-tem­per­ature monoclinic to a disordered rhombohedral structure are observed in Na_3_Sc_2_(PO_4_)_3_ (NSP). In spite of numerous investigations, the structural phase relationships are still surprisingly an enigma. At tem­per­atures above 500 K, the com­pound shows the γ-structure with *R* 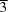 *c* symmetry. Two phase changes at about 340 K (monoclinic α → rhombohedral β) and 440 K (β → superionic rhombohedral γ) have been reported in polycrystalline materials (Boehm *et al.*, 1981 ▸; Delbecq *et al.*, 1981 ▸; Boilot *et al.*, 1981 ▸). However, samples synthesized under different conditions, as well as single-crystalline *versus* polycrystalline material, seem to behave differently, and three different space groups have been suggested for the room-tem­per­ature (α) modification (Kim *et al.*, 2017 ▸; Moon *et al.*, 2016 ▸; Guin *et al.*, 2017 ▸; Wang *et al.*, 2016 ▸), making the situation even more opaque.

The mobility and site preference of alkali-metal ions are key to understanding the phase-transition sequences. Even small site preferences and small occupations of possible sites within the voids of the framework can reduce symmetry. Nevertheless, an accurate study on the interplay of the site preference and the phase behaviour is limited by the low X-ray scattering intensity of Li^+^ and Na^+^.

In order to shed light on this interplay, we replaced Na^+^ by the much heavier Ag^+^ using an ion-exchange reaction. This ion exchange allows for the investigation of the crystal structure and its tem­per­ature dependence between 100 and 520 K by means of X-ray diffraction.

## 2. Experimental   

### 2.1. Synthesis   

The polycrystalline Na_3_Sc_2_(PO_4_)_3_ sample material was syn­thesized using a ceramic sintering process and was used to evaluate the extent and speed of Na^+^–Ag^+^ ion exchange. The exchange experiments on the single crystals were then designed with these results in mind. Following the procedure proposed by Collin *et al.* (1986 ▸), a stoichiometric mixture of 3 moles NaPO_3_ and 1 mole Sc_2_O_3_ (lab-code HB) was carefully ground and initially heated in a muffle furnace at 673 K for 4 h to decom­pose the starting materials. The obtained material was ground again in an agate mortar, pelletized and reheated in open platinum crucibles in a resistance furnace to 1573 K at a rate of 100 K h^−1^. The sample was kept at this tem­per­ature for 20 h, after which the furnace was turned off. The sample was left to cool in the furnace until it reached room tem­per­ature.

Large single crystals of NSP were synthesized using the two-step process described by Collin *et al.* (1986 ▸) and Sotofte & Fu (1988 ▸). In the first step, phase-pure ScPO_4_ was synthesized from 0.5 moles Sc_2_O_3_ and 1 mole (NH_4_)H_2_(PO_4_) by preheating the mixture at 673 K for 4 h, regrinding, pelletizing and then annealing at 1473 K for 20 h. Single crystals were grown using the flux method: 1 mole ScPO_4_ was mixed with 6 moles Na_4_P_2_O_7_ as the high-tem­per­ature flux. The mixture was placed in a platinum crucible, covered with a lid, heated to 1373 K at a rate of 100 K h^−1^ and held at this tem­per­ature for 5 h to allow the melt to homogenize. The sample was then cooled slowly to 1173 K at a rate of 3 K h^−1^, before final cooling to room temperature at a rate of 450 K h^−1^ (furnace cooled). The solidified melt was washed several times in hot water to dissolve the flux and to obtain single crystals, which had good cuboid shapes of up to 0.5 mm in size.

The Ag_3_Sc_2_(PO_4_)_3_ (ASP) sample material (poly- and single crystalline) was obtained *via* ion exchange from Na_3_Sc_2_(PO_4_)_3_. For this, 250 mg of the as-grown polycrystalline NSP sample material was immersed in 50 ml of a 1 molar aqueous AgNO_3_ solution for various lengths of time to observe the rate of Na^+^–Ag^+^ exchange. Based on these results, the single crystals were then immersed in the same com­position solution for 12 h to guarantee full Na^+^–Ag^+^ exchange.

### 2.2. Scanning electron microscopy (SEM)   

Crystallite morphology and semiquantitative chemical analyses were evaluated by means of Field Emission Scanning Electron Microscopy (FE-SEM) using a Zeiss Ultra Plus II microscope coupled with an Oxford Instruments X-MAX 50 mm^2^ SDD Energy Dispersive X-ray spectrometer (EDX). Samples were mounted on standard Al-sample holders with adhesive graphite tabs and coated with carbon to enhance electric conductivity. Synthesis materials were analysed at different magnifications with a 20 kV acceleration voltage and working distances of 4–9 mm (using SE or InLens detectors). The EDX analyses (mapping) were performed to confirm com­plete Na^+^ –Ag^+^ replacement.

### 2.3. Thermal analysis   

Low-tem­per­ature caloric measurements were made at Salzburg University by E. Dachs on a Quantum Design Physical Property Measurement System (PPMS) equipped with the relaxation calorimeter option of the PPMS system. Data were collected three times (and averaged for each step) at 180 different tem­per­atures between 2 and 300 K, using a logarithmic spacing with increasing data density toward lower tem­per­atures. Additional *C*_p_ data sets were collected between 151 and 302 K in 1 K steps. More details on the PPMS technique can be found in the literature (Dachs & Bertoldi, 2005 ▸; Dachs & Benisek, 2011 ▸). High-tem­per­ature heat capacity was recorded between 250 and 573 K by A. Benisek (Salzburg University) in a step scanning mode with a power-com­pensated PerkinElmer Diamond Differential Scanning Calorimeter (DSC). The DSC heat capacity measurements were carried out under an argon flow during heating and cooling, with a heating rate of 10 K min^−1^. A single crystal of corundum was measured as a calibration standard. For more details on measurements and calibration, see, for example, Benisek *et al.* (2010 ▸) and Dachs *et al.* (2012 ▸, 2018 ▸).

### 2.4. Powder X-ray diffraction (PXRD)   

PXRD data were collected at room tem­per­ature in coupled Θ–Θ mode on a Bruker D8 Advance with a DaVinci Design diffractometer equipped with a solid-state Lynxeye detector and an automatic sample changer. Data were acquired using Cu *K*α_1,2_ radiation between 10 and 90° 2Θ, with a step size of 0.01°, an integration time of 1 s and with the divergence slit and the receiving slits opened at 0.3 and 2.5°, respectively. A primary and secondary side 2.5° Soller slit was used to minimize axial divergence and a detector window opening angle of 2.95° was chosen. Refinement of the lattice parameters from PXRD data collected on samples from time-dependent Na^+^–Ag^+^ exchange experiments was performed with *TOPAS* (Version 4.2; Bruker, 2012 ▸). For Rietveld analyses, the background was modelled with a Chebychev function of 10th order and the fundamental parameter approach was used to describe the peak shape of the Bragg reflections. The structural models applied to Na_3_Sc_2_(PO_4_)_3_ were taken from Collin *et al.* (1986 ▸). A model was determined from the single-crystal X-ray diffraction data for Ag_3_Sc_2_(PO_4_)_3_ in this study as the data from the literature (Atovmyan *et al.*, 1983 ▸) did not match well.

### 2.5. Single-crystal X-ray diffraction   

Single-crystal X-ray diffraction data were collected on a Bruker SMART APEX CCD diffractometer. The single crystals were selected on the basis of their optical properties (sharp extinctions, regular shape and homogeneity in colour) and glued on top of a glass capillary (0.1 mm in diameter). Intensity data were collected on four different crystals at room tem­per­ature with graphite-monochromated Mo *K*α X-ray radiation (50 kV, 20 mA). The crystal-to-detector distance was 30 mm and the detector was positioned at −28° 2θ and −40° 2θ using an ω-scan mode strategy at four different φ positions (0, 90, 180 and 270°). A total of 666 frames with Δω = 0.3° were acquired for each run. Low-tem­per­ature data sets were recorded down to tem­per­atures between 300 and 90 K using a Bruker Cryojet evaporating low-tem­per­ature device with liquid nitrogen as the cooling medium. Two data sets (300 and 520 K) were collected at the TU Dresden on a Bruker AXS Kappa APEXII CCD diffractometer with graphite-monochromated Mo *K*α X-ray radiation (50 kV, 30 mA), equipped with a standard calibrated Nonius FR559 Crystal Heater (Tuinstra & Storm, 1978 ▸). The crystal-to-detector distance was 50 mm and the detector was positioned at 2θ positions of 30 and 45° for the measurements using an ω-scan mode at four different φ positions (0, 90, 180 and 270°).

3D data were integrated and corrected for Lorentz, polarization and background effects using *APEX3* software (Bruker, 2015 ▸). Structure solutions, using direct methods and subsequent weighted full-matrix least-squares refinements on *F* ^2^, were carried out with *SHELX2012* (Sheldrick, 2015 ▸), as implemented in the program suite *WinGX2014.1* (Farrugia, 2012 ▸). Experimental data are given in Table 1 ▸.

## 3. Results and discussion   

### 3.1. Synthesis and exchange experiments   

Single-phase polycrystalline NSP was obtained in the single-step synthesis at 1573 K. The as-synthesized NSP samples are white with a very pale-blue tint. Indexing of the PXRD data yields a monoclinic cell [*a* = 15.7168 (4), *b* = 8.9328 (1) and *c* = 9.0408 (2) Å, and β = 126.035 (3)°] that can be most accurately structurally refined with *C*2/*c* symmetry, as proposed pre­viously (Guin *et al.*, 2017 ▸; Redhammer *et al.*, 2020 ▸; Ladenstein *et al.*, 2020 ▸). The recognition of a monoclinic cell at room tem­per­ature contrasts with findings in the recent literature where more general *R* 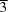 *c* (Liu *et al.*, 2018 ▸) symmetry is reported.

**Figure 1:**
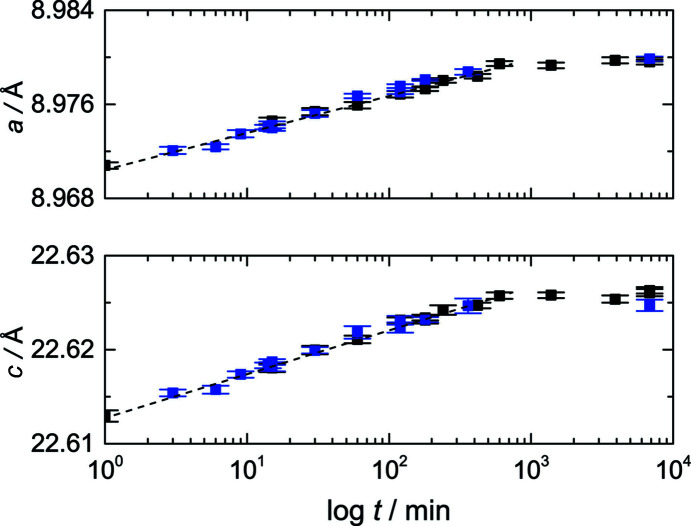
The change of lattice parameters *a* and *c* with immersion time of Na_3_Sc_2_(PO_4_)_3_ polycrystalline material in 1 molar AgNO_3_ solution.

The NSP became pale-yellow immediately on coming into contact with the AgNO_3_ solution due to the Ag^+^–Na^+^ exchange reaction. The PXRD data clearly indicate that the main portion of NSP transforms to ASP within a period of 1 min of immersion in the Ag-bearing solution (see Fig. S1 in the supporting information). The diffraction pattern of ASP can be indexed on the basis of a trigonal *R* 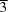 *c* unit cell and Rietveld refined with *a* = 8.9708 (3) and *c* = 22.6129 (6) Å. Only minor amounts of an impurity phase, *i.e.* Ag(PO_4_)_3_, were observed. The *a*-lattice parameter increases by about 0.1% (∼0.009 Å) as immersion times extend beyond 1 min. After 10 h of treatment, only a minor increase can be observed that follows a simple power law, as shown in Fig. 1 ▸(*a*). Similar observations are made for the *c*-lattice parameter, *i.e.* within 1 min, the lattice parameter increased by about 0.06% (∼0.0135 Å) and no significant change can be observed after 10 h (*cf*. Table S1 in the supporting information).

Comparison of the lattice parameters of untreated NSP with those of ASP highlights the fact that the replacement of Na^+^ (*r* ^IV^ = 1.02 Å) by Ag^+^ (*r* ^IV^ = 1.15 Å) mainly affects the *c*-lattice parameter and expands it by 1.6%, while the *a*-lattice parameter is increased by only 0.5%. The replacement of Na^+^ by Ag^+^ thus has distinct effects on, and results in rearrangements in, the Sc_2_(PO_4_)_3_ framework.

**Figure 2:**
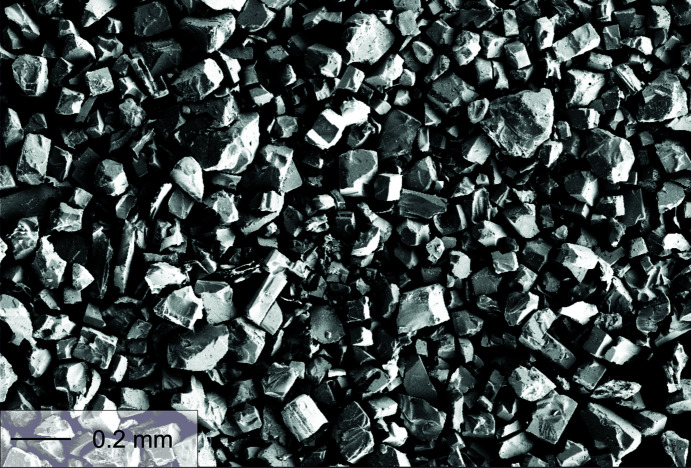
Secondary electron image, taken on a scanning electron microscope (SEM), showing the typical morphology of the Ag_3_Sc_2_(PO_4_)_3_ crystals, which were obtained from a flux-grown Na_3_Sc_2_(PO_4_)_3_ material *via* ion exchange. EDX spot analyses and mapping on the surface and on cross sections of some selected crystals (cut in the middle and cutting faces analysed) yield no indications of Na^+^, but rather a homogenous distribution of Ag^+^ on and throughout the crystals. Details of these measurements are reported in Ladenstein *et al.* (2020 ▸).

Based on the above observations of a very fast ion-exchange reaction, a leaching time of 12 h was taken to be sufficient for com­plete ion exchange in the single crystals. The highly transparent and cuboid-shaped crystals do not show any morphological change due to the Na^+^–Ag^+^ exchange, *i.e.* no cracks or defoliation of the crystals are observed. A secondary electron image of typical exchanged ASP crystals is shown in Fig. 2 ▸. The single crystals are cube-shaped with mostly well-developed faces. They average around 100 µm in size with individual crystals up to 300 µm.

### 3.2. Thermal analysis   

**Figure 3:**
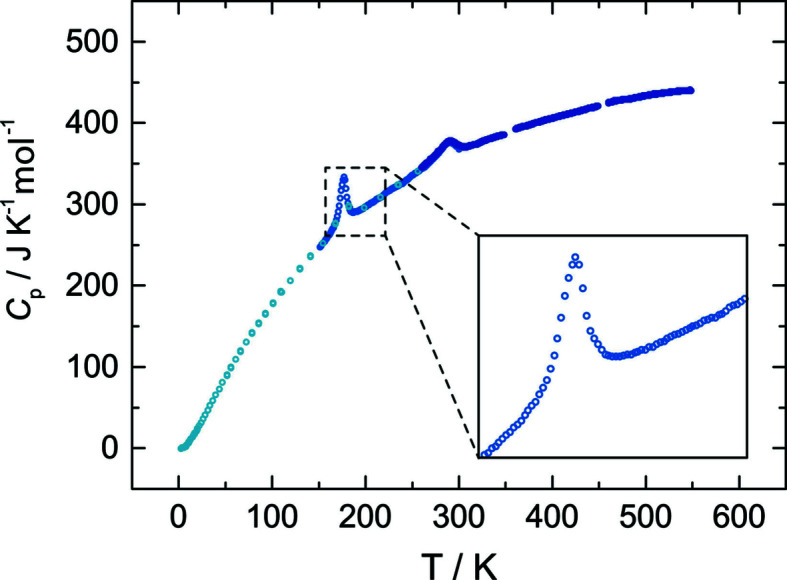
Heat capacity of small single crystals of Ag_3_Sc_2_(PO_4_)_3_ between 2 and 550 K. The inset depicts the heat capacity of the polycrystalline sample.

Heat capacity measurements (Fig. 3 ▸) on a small amount (27 mg) of unground single crystals reveal two events in the tem­per­ature evolution: one very prominent peak centred at 177 (1) K and a second, rather broad, peak at 292 (1) K. PPMS (low tem­per­ature) and DSC measurements correlate well in the overlapping tem­per­ature range. The two peaks correspond to two structural phase transitions (see below). A DSC run on a larger sample mass of polycrystalline ASP (immersion time 6 h) revealed, however, a somewhat different behaviour (see inset in Fig. 3 ▸). Here, the peak at around room tem­per­ature is much smaller and is shifted towards 304 K. Nevertheless, all the observed peaks exhibit a lambda (λ) shape, typical for order–disorder transitions (Benisek *et al.*, 2018 ▸; Benisek & Dachs, 2018 ▸). The phase transition energy, determined by integrating the area beyond the peaks, amounts to 739 (3) J g^−1^ for the transition at 177 K and 445 (5) J g^−1^ for the 304 K transition. As the phase transitions are associated with an increase in tem­per­ature in the sample material, the reactions are endothermic and the high-tem­per­ature phases having a higher entropy are thus more disordered and have a higher symmetry.

### 3.3. Structural characterization   

#### 3.3.1. The high-tem­per­ature γ-form   

**Figure 4:**
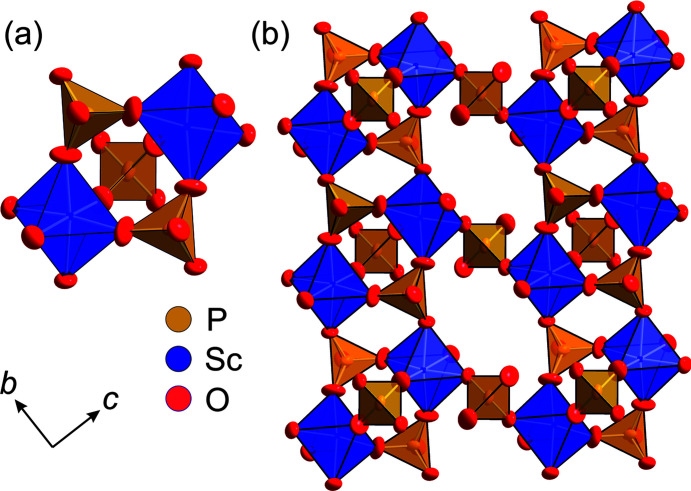
Polyhedral drawings of the structure of NASICON-type com­pounds. Alkali-metal positions have been omitted to highlight the 3D skeleton structure. (*a*) The basic lantern unit and (*b*) a section of the Ag_3_Sc_2_(PO_4_)_3_ *R* 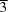 *c* structure, viewed along [100]. Anisotropic atomic displacement parameters are drawn at the 95% probability level.

At room tem­per­ature, the observed single-crystal X-ray diffraction intensity data can be indexed on the basis of a rhombohedral unit cell, with *a* = 8.9756 (4) and *c* = 22.6248 (10) Å, and analysis of the systematic extinctions yields the space group *R* 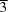 *c* (No. 167), with *Z* = 6. Structure solutions using direct methods (Sheldrick, 2015 ▸) and charge-flipping methods, as applied in the *JANA2006* software (Petříček *et al.*, 2014 ▸), converged on one structural model, which is very similar to the commonly observed skeleton structure of γ-type NaSICON com­pounds (*e.g.* Redhammer *et al.*, 2016 ▸; Monchak *et al.*, 2016 ▸, and references therein). The octahedrally coordinated Sc^3+^ are in the 12*c* position (site symmetry 3.), the tetrahedrally coordinated P^5+^ in the 18*e* position (site symmetry 0.2) and the two independent O atoms in general 36*f* positions. Two ScO_6_ octa­hedra, which share all their corners with PO_4_ tetrahedra, and three PO_4_ tetrahedra, sharing all their corners with neighbouring ScO_6_ octahedra, build a basic repeat unit with an [Sc_2_(PO_4_)_3_]^3−^ com­position, which is known as the lantern unit (Masquelier & Croguennec, 2013 ▸) and is depicted in Fig. 4 ▸(*a*). Each lantern unit is connected to six others, thus generating a 3D framework with large interstitial voids that accommodate the Ag^+^ (or more generally the alkali) cations. These lanterns are stacked parallel to the [001] direction within the common rhombohedral structure space group *R* 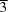 *c*.

For Na_3_Sc_2_(PO_4_)_3_, Collin *et al.* (1986 ▸) reported a disorder of the tetrahedra with highly anisotropic vibration amplitudes of the P cations and also for the anions. In addition, they observed broad Raman bands for PO_4_ modes. From this, they concluded that the rotational terms for the PO_4_ tetrahedra exist and result in some positional disorder. For ASP in the γ-phase, the Sc atoms show highly isotropic displacement parameters (*U*_max_ to *U*_min_ of the principal mean-square atomic displacement are 0.0088 and 0.0073 Å^2^, respectively, at 300 K). Indeed, the P atoms display some higher anisotropic vibration (*U*_max_ = 0.0149 Å, *U*_min_ = 0.0089 and *U*_max_/*U*_min_ ≃ 2.4), which could be interpreted as a sign of some minor positional disorder. Increasing the tem­per­ature to 500 K increases the overall size of the thermal vibration, while the anisotropy is somewhat reduced, with *U*_max_/*U*_min_ ≃ 2.2. The O atoms show larger anisotropic atomic displacement, with *U*_max_/*U*_min_ of 3.8 and 2.4 for the O1 and O2 atoms, respectively. As expected, ADP_max_ values are perpendicular to the *M*—O—P bonds.

**Figure 5:**
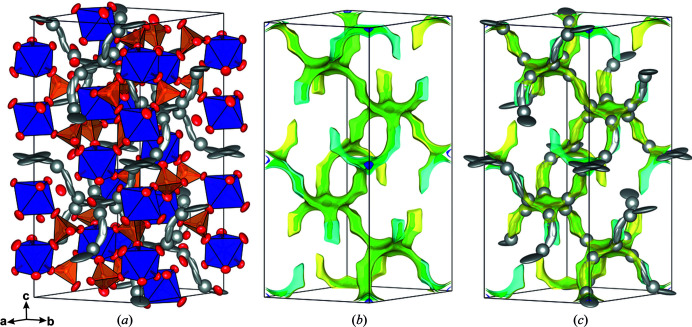
(*a*) The Ag_3_Sc_2_(PO_4_)_3_ structure at 300 K in the *R* 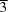 *c* phase, with anisotropic atomic displacement ellipsoids at the 95% probability level, com­pared to the calculated bond-valence electron landscape maps [at the 0.02 (dark blue), 0.20 (green) and 0.45 eV (yellow) level above minimum] (*b*) without and (*c*) with the experimentally obtained Ag^+^ positions.

In the γ-phase, the Ag^+^ cations are reported to occupy two different cavities: *M*1 is at the 6*b* position (0,0,0) with site symmetry 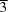 and corresponds to an empty octahedral site in the cubic close-packed arrangement of corundum. The *M*2 site is located at the 18*e* position (*x*, 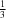, 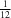) (Catti *et al.*, 2004 ▸). However, some slight displacements to the general 36*f* positions are observed (Redhammer *et al.*, 2016 ▸). In ASP, the Ag^+^ ions are distinctly disordered within the cavities of the framework. The normal 6*b* *M*1 site turned out to be unoccupied during refinement and the Ag^+^ ions are shifted mainly along *y* away from the 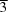 axis to the general position 36*f* at [0.017 (2), 0.0807 (6), 0.0019 (2)], which is denoted as the Ag11 site. It is still close to the 6*b* position and has an occupancy of 1.012 (7) Ag^+^ per formula unit (pfu). The electron density around the 18*e* position is best modelled by a split model with two positions: the regular 18*e* position Ag2, which is located at the twofold axis and is occupied by 1.14 (1) Ag^+^ pfu, and a position denoted Ag22 that is displaced from Ag2 towards the 6*b* site by about 0.55 Å and is occupied by 0.84 (1) Ag^+^ pfu. However, the best-fit model for the observed electron density requires a further ‘site’ for Ag^+^ between the Ag22 and Ag11 positions. Due to the low occupancy [0.052 (6) pfu], only isotropic displacement parameters could be refined. For all other Ag^+^ sites, full anisotropic refinement of the atomic displacement parameters is possible and yields highly anisotropic cigar-shaped vibration ellipsoids, which are oriented along the most probable ion-diffusion path and partly overlap each other, as can be seen in Fig. 5 ▸(*a*). This indeed suggests a more or less continuous distribution of Ag^+^. Similar observations were made by Atovmyan & Tkachev (1995 ▸); however, their Ag positions do not exactly match those found herein and, applying their structural model, we were neither able to reproduce our powder nor our single-crystal intensity data. Regardless of the observed differences, the above authors observed a displacement of Ag^+^ from *M*1 at 6*b*, as well as from the regular *M*2 position. Moreover, the ions are less disordered, as found in our study. In summary, the site-occupation factors indicate 3.03 Ag^+^ per formula unit, which is very close to the expected value.

For the 298 K data set, refinements with anharmonic anisotropic atomic displacement parameters were also performed using the *JANA2006* software. Using third-order (Ag11 and Ag2) and fourth-order (Ag22) anharmonic anisotropic displacement parameters (ADPs), com­parably low reliability factors are obtained without needing the intermediate Ag12 ‘site’. The obtained joint probability density functions of both refinement models are very similar to each other, but those with harmonic ADPs appear to be smoother and thus the harmonic model is preferred here.

**Figure 6:**
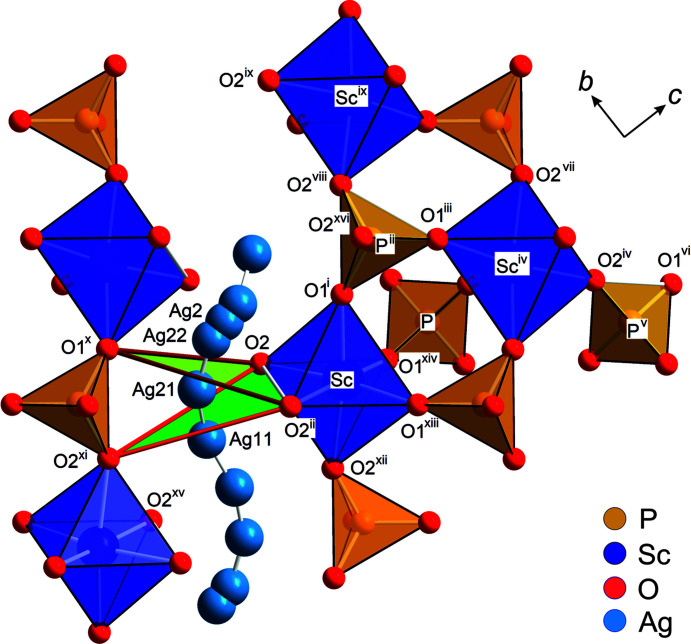
Part of the NaSICON structure of Ag_3_Sc_2_(PO_4_)_3_ at 298 K (*R* 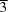 *c*), viewed along [100], showing a lantern unit and some neighbouring polyhedra for γ-ASP. The atom nomenclature and equivalent atoms are as in the text. [Symmetry codes: (i) −*y* + 1, *x* − *y*, *z*; (ii) −*y*, *x* − *y*, *z*; (iii) *y* − 1, *x*, −*z* + 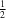; (iv) *x* − *y*, −*y*, −*z* + 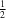; (v) −*x* + 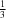, −*y* − 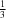, −*z* + 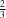; (vi) −*x* + 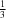, −*y* + 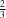, −*z* + 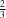; (vii) *y*, *x*, −*z* + 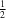; (viii) −*y* + 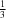, −*x* + 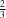, *z* + 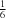; (ix) −*x* + *y* + 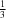, *y* + 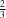, *z* + 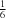; (x) −*x* + 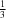, −*x* + *y* − 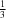, −*z* + 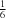; (xi) *x* − *y*, *x*, −*z*; (xii) −*x* + *y*, −*x*, *z*; (xiii) −*x* − 1, −*x*, *z*; (xiv) *x*, *y* − 1, *z*; (xv) *y*, −*x* + *y*, −*z*; (xvi) *y* − 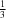, −*x* + *y* + 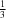, −*z* + 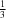.]

Alkali-metal ions can jump between two adjacent sites by bypassing a bottleneck. The bottleneck is defined by two triangles, which have one common *M*O_6_ edge and expand to the O1 and O2 atoms of a tetrahedron opposite to the *M*O_6_ octahedron (Kohler & Schulz, 1985 ▸) (*cf* Fig. S2 in the supporting information). A measure of the size of this bottleneck is the area of the two triangles T1 (*e.g.* O2—O2^ii^—O1^x^; see Table 2 ▸ for symmetry codes i–xv) and T2 (*e.g.* O2—O2^ii^—O2^xi^), as illustrated in Fig. 6 ▸. There is a direct correlation between the area and the activation energy for the jump processes, denoted *E*_a_ (Guin & Tietz, 2015 ▸; Losilla *et al.*, 2000 ▸). For ASP, the T1 and T2 areas at room tem­per­ature are 5.495 and 5.952 Å^2^, respectively. These are at the upper end of the range com­piled by Guin & Tietz (2015 ▸) for Na-bearing NaSICON-type com­pounds, suggesting that the substitution of Na^+^ by Ag^+^ opens up the structure and, therefore, also the bottlenecks.

There is direct evidence in the room-tem­per­ature structural data that the 3D ion-diffusion pathway is through these bottlenecks. Considering the shortest Ag^+^ jump distances, the pathway is along …Ag11–Ag11–Ag21–Ag22–Ag2–Ag22–Ag21…, with jump distances of 0.667 (3)–1.352 (2)–1.186 (2)–0.500 (2)–0.500 (2)–1.186 (2) Å, respectively. Interestingly, this diffusion pathway bypasses the regular *M*1 and *M*2 sites and allows very short jump distances. The experimentally observed diffusion pathways are further confirmed by bond-valence electron landscape (BVEL) map calculations (Katcho *et al.*, 2019 ▸) (see Fig. 5 ▸). From these BVEL maps, the activation energies for Ag^+^ diffusion along the different crystallographic sites are estimated to be below 0.2 eV.

To test for any changes in Ag^+^ distribution, part of the sample material was annealed for 72 h at 573 K and rapidly quenched to room temperature. Analysis of the intensity data from three experiments at this annealing tem­per­ature yield very similar results (see Table S2 in the supporting information for details). Thus, the distinct disorder of the Ag^+^ ions is not removed even at a low annealing tem­per­ature, when cationic ordering is usually reached. Hence, the results highlight this intrinsic property of the *R* 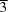 *c* structure of ASP.

*In situ* measurements at 520 K reveal no significant changes to the distribution of Ag^+^; only a slightly higher occupancy of the Ag21 position at the expense of Ag2 is observed (see Table S2 in the supporting information). During an increase in tem­per­ature, the Sc—O1^i^ and Sc—O2 bond lengths expanded somewhat (Table 2 ▸ and Table S3 in the supporting information), causing the average 〈Sc—O〉 to increase from 2.090 (2) to 2.094 (2) Å, the polyhedral volume to increase and the bond-angle distortion to be reduced. For the PO_4_ tetrahedra, a small reduction of the bond lengths by ∼0.004 Å is observed with increasing tem­per­ature, but the polyhedral distortion parameters remain very similar at room temperature and 520 K (Table S3 in the supporting information). A shortening of the P—O distances in NaSICON-type com­pounds has already been described by Kohler & Schulz (1985 ▸) in their study of NZSP com­pounds. Generally, alterations in structural parameters within the first coordination sphere around Sc and P are small; however, there are some more evident variations within the second coordination sphere, *i.e.* within the con­nection of individual polyhedra. The lantern unit is stretched parallel to the *c* direction, expressed by an increase of the Sc—Sc^iv^ distance from 4.602 (2) to 4.622 (2) Å (Fig. 6 ▸). This is facilitated by an increase of the Sc—O1^i^ bond length and a slight closing of the intra-lantern *M*—O1—P angle, *e.g.* the Sc—O1^i^—P^ii^ in Fig. 6 ▸. These changes directly influence the *c*-lattice parameters and are responsible for the distinct increase with tem­per­ature.

The inter-lantern *M*—O2—P angle (*e.g.* Sc^iv^—O2^iv^—P^v^ in Fig. 6 ▸) increases with tem­per­ature by 0.9 (1)°. The rotation of the M and P polyhedra is linked to an opening of the bottleneck and an increase in the inter-lantern O1⋯O2 and O1⋯O2 distances. Additionally, this is achieved by an opening of the O2—Sc—O2^ii^ octahedral bond angle and an increase of the O2–O2^ii^ octahedral edge by ∼0.014 (2) Å. At 520 K, the T1 and T2 areas are 5.555 and 6.071 Å^2^, respectively, reflecting the higher ionic conductivity with increasing tem­per­ature, as observed previously (Ladenstein *et al.*, 2020 ▸).

The 520 K data for ASP can be directly com­pared to NSP, which also has an *R* 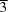 *c* structure at 500 K (Ladenstein *et al.*, 2020 ▸). One distinct difference is that NSP contains a more com­pressed lantern unit along the *c* direction, with an Sc⋯Sc^iv^ separation of 4.556 (2) Å, which is ∼0.066 (2) Å less than in ASP. The intra-lantern *M*—O1—P angle (Sc—O1^i^—P^ii^) is also distinctly lower by as much as 1.85 (5)° (*cf* Table S3 in the supporting information). Furthermore, within the ScO_6_ octahedron, significant changes are induced by the Na^+^–Ag^+^ exchange: the Sc—O2 bonds are evidently longer in ASP com­pared to NSP [2.133 (1) and 2.112 (2) Å at 520 and 500 K, respectively]. Moreover, the Sc—O1 bonds are more elongated, which leads to an increase of <Sc—O> by 0.013 (2) Å, an increase of the polyhedral volume, a slight increase in the polyhedral distortion index and a lower, *i.e.* more regular, bond-angle configuration in ASP. For the tetrahedral site, the P—O distances are more similar to each other than Sc—O, but again the P—O2 bond lengths are noticeably longer by 0.015 (3) Å in ASP, while the P—O1 bond lengths remain constant [−0.005 (3) Å]. Overall, the PO_4_ tetrahedron in ASP appears to be similar to that in NSP, with a slightly larger polyhedral volume, a larger distortion index and a lower bond-angle variance. The *M*—O2—P inter-lantern angle is not affected by the Na^+^–Ag^+^ substitution. Nevertheless, the construction of the octahedral–tetrahedral connection changes with the Sc^iv^—O2^iv^—O1^vi^ angle, which involves the O2^iv^–O1^vi^ tetrahedral edge. The decrease in this angle from ASP to NSP of 1.1° has a direct influence on the bottleneck size for alkali-ion migration; it is more closed in NSP, with T1 and T2 areas of 5.384 and 5.598 Å^2^, respectively, at 500 K. These values com­pare well with those given in Guin & Tietz (2015 ▸) and allow fast ion movement with low activation energies.

#### 3.3.2. The intermediate *C*2/*c* β-form   

**Figure 7:**
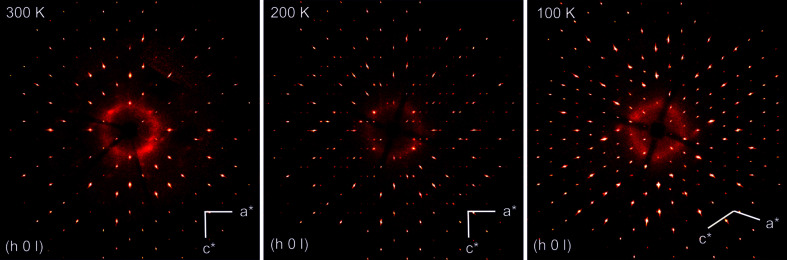
Calculated precession images of the (*h*0*l*) plane of Ag_3_Sc_2_(PO_4_)_3_ at tem­per­atures of 300, 200 and 100 K, supporting a structural phase transition between these tem­per­atures.

ASP undergoes a phase transition between 300 and 270 K, as is shown by the calculated precession images of the (*h*0*l*) plane (see Fig. 7 ▸). This polymorph is preserved down to 180 K.

Indexing of all the observed intensity data is possible using a tri-twinned monoclinic unit cell, with *a* = 15.5374 (2), *b* = 8.9703 (1) and *c* = 22.5718 (3) Å, and β = 89.998 (4)° at 200 K. The unit-cell parameters of the monoclinic and rhombohedral cells are related to each other by 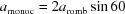
°, 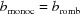 and 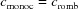. Intensity statistics and systematic extinctions provide strong evidence for the space group *C*2/*c*, with *Z* = 4. Structure solution yields a structural model with a framework very similar to the ordered low-tem­per­ature structure reported for Na_3_V_2_(PO_4_)_3_ by Chotard *et al.* (2015 ▸). It should be noted that full indexing of the data can also be achieved on the basis of a trigonal cell, with *a* = *b* = 18.060 (8) and *c* = 22.661 (11) Å, in the space group *P* 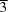 *c*1; however, no definitive structure solution was possible. As outlined in more detail in the supporting information, models assuming (in)commensurate modulations were also tested, but did not yield satisfactory results and it is thus assumed that tri-twinning best describes the observed data. This is a very similar situation to the case of NSP (Ladenstein *et al.*, 2020 ▸) and details on this com­pound are given in a com­panion paper (Redhammer *et al.*, 2020 ▸).

The monoclinic β-form of ASP shows three independent Sc-atom positions, five independent P-atom positions and 18 independent O-atom positions, all of them on general positions 8*f*, except for P5, which resides at a 4*e* Wykoff position. The individual ScO_6_ octahedra are similar to each other in the β-form, with 〈Sc—O〉 bond lengths of 2.095, 2.094 and 2.088 Å at 270 K. There are also no significant differences in the volume and polyhedral distortion indices. The Sc1O_6_ octahedron has the largest average Sc—O bond length and appears to be more regular than the others. No significant changes occur within the first coordination sphere of Sc^3+^ during transition from the β- to the γ-form. In NSP, the three individual octahedra have almost the same 〈Sc—O〉 bond lengths and polyhedral volumes at 300 K. However, a significant bond-angle variance leads to a polyhedral distortion (see Table S3 in the supporting information). Hence, the replacement of the smaller Na^+^ ion by the larger Ag^+^ ion yields more regular local environments around Sc. The bond angles narrow with decreasing tem­per­ature from 270 to 180 K. The five PO_4_ tetrahedra are mutually similar in size and distortion, with 〈P—O〉 values ranging between 1.529 and 1.535 Å, whereby the P1 and P4 tetrahedra appear to be the largest and smallest, respectively. The tetrahedra are remarkably regular, with smaller distortion indices and a small bond-angle variance (Table S3 in the supporting information). In addition, there is almost no change to the tetrahedron observed within the stability range of the β-phase. Similar to the octahedral sites, the average 〈P—O〉 bond lengths of the tetrahedral site are larger for ASP (about 0.008 Å) com­pared to NSP at 270 and 300 K. Moreover, the slightly smaller 〈P—O〉 distances invoke larger bond-angle variances in NSP.

The overall topology of the β-form is closely related to the γ-form. The interconnection of individual polyhedra, *i.e.* between the three distinct ScO_6_ octahedra and the five distinct PO_4_ tetrahedra, which allows for a larger degree of freedom for structural adjustments, largely resembles the γ-phase. Two different lantern units can be discerned: one is built up by two Sc1O_6_ octahedra, two P2 tetrahedra and one P4 tetrahedron, whereas the second consists of Sc2O_6_ and Sc3O_6_ octahedra, and P1, P3 and P5 tetrahedra. Within these lantern units, the *M*—O—P angles range between 152.8 (2) and 153.7 (2)° at 180 K, with an average value of 153.3 (2)°, which is similar to that at 300 K in the γ-phase. There are no trends evident with increasing tem­per­ature: the distance between two next-neighbour Sc1 atoms is 4.5910 (13) Å at 180 K and increases slightly to 4.6020 (12) Å at 270 K; the Sc2⋯Sc3 separation is smaller and varies between 4.5897 (11) and 4.5930 (14) Å, with no clear tem­per­ature dependence, but increases to 4.5977 (8) Å during the phase transition; and the inter-lantern unit *M*—O—P angles all range between 145.7 (2) and 146.5 (2), *i.e.* within the estimated standard deviations. Hence, there is neither a clear variation with tem­per­ature nor a significant alteration in the *M*—O—P angles. The structure of ASP at 270 K can be com­pared with that of NSP at 300 K. Both com­positions are in the *C*2/*c* β-structural state. The Sc—O bond lengths are larger in the high-tem­per­ature ASP structure and the polyhedral distortion is distinctly smaller, which is most prominent in the bond-angle variance (Table S3 in the supporting information). The same is true for the PO_4_ tetrahedra, where 〈P—O〉 distances are ∼0.008 Å longer on average in ASP. It is evident that the variation in the Sc⋯Sc distances within the lantern units, as well as the intra- and inter-lantern unit *M*—O—P angles, are ∼0.1 Å smaller in NSP. Hence, the lantern units in NSP are more com­pressed and that leads to a distinctly smaller *c*-lattice parameter.

**Figure 8:**
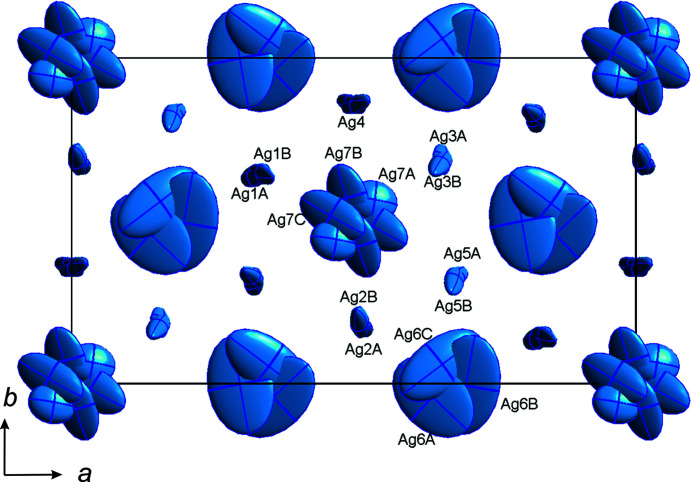
Displacement ellipsoid plot (95% probability level) for the Ag^+^ ions in Ag_3_Sc_2_(PO_4_)_3_ at 200 K viewed down [001].

Using a similar approach to refine ASP as used for the NSP *C*2/*c* β-phase (Ladenstein *et al.*, 2020 ▸), it turns out that an adequate modelling of alkali ions is only possible by using split atomic positions for Ag1 to Ag5 (except for Ag4, which is close to a 4*e* special position and, thus, displays a split character by nature) to describe the highly anisotropic behaviour of the Ag atoms. High densities are evident on residual electron-density maps [with the refined Sc_2_(PO_4_)_3_ framework and the Ag1–Ag5 positions] that form a highly anisotropic ‘star’-like shape centred around 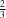, 0, 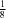 (Ag6) and 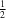, 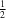, 0 (Ag7). Modelling of this pattern was only possible by splitting both positions into three com­ponents, indicating a highly anisotropic nature of the Ag^+^ occupation around the entire Ag6 and Ag7 positions (see Fig. 8 ▸). All positions other than Ag6 are only partly filled: the Ag1–Ag5 positions are filled by about 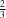 and the Ag7 position is 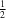 filled. In this work, the occupation of all the split positions were then added together to describe the entire site. Generally, the Ag^+^ ions in the β-phase are more centred in their positions than in the γ-phase, where there is a more continuous Ag^+^-ion distribution. Interestingly, the refined site occupations do not show any significant change with tem­per­ature. The total Ag^+^ occupation is 3.02 (1) Ag^+^ pfu, which is similar to that obtained for the γ-phase.

Bottleneck sizes between octahedral edges and opposite tetrahedra vary significantly due to the larger number of distinct Sc and P sites. Since the bottleneck size affects ion mobility, and ion mobility is related to the ordering phenomena in NaSICON materials, slightly different order–disorder transition tem­per­atures can be expected, which explains the broad peak observed in the DSC data. Moreover, as tem­per­ature increases, the bottlenecks open up and Ag^+^ disorder increases, leading to a phase transition to the γ-phase at 300 K. Possible Ag^+^–Ag^+^ jump distances are significantly larger and range between 2.63 (14) and 2.862 (12) Å for Ag^+^ ions in distinct sites, and between 0.618 (18) and 0.899 (17) Å within the split positions of a specific site (see Table 2 ▸).

#### 3.3.3. The low-tem­per­ature *C*2/*c* α-form   

It is evident from the heat capacity *C*_p_ measurements that a phase transition occurs around 177 K. Indeed, simulated precession images clearly show that the structure has changed between 180 and 150 K (see Fig. 7 ▸ *c*).

**Figure 9:**
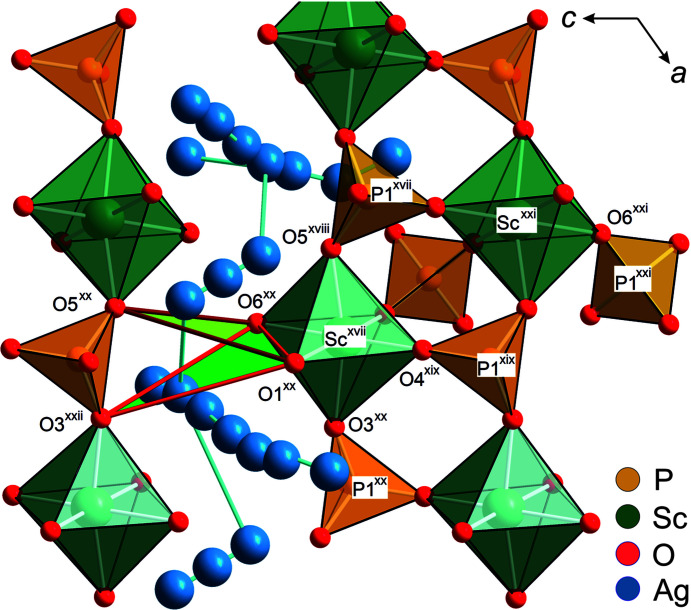
Part of the NaSICON structure of Ag_3_Sc_2_(PO_4_)_3_ at 298 K (*C*2/*c*), viewed down [010], showing a lantern unit and some neighbouring polyhedra for α-ASP. The atom nomenclature and equivalent atoms are as used in the text. [Symmetry codes: (xvii) *x*, *y*, *z* − 1; (xviii) −*x* + 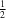, −*y* + 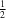, −*z*; (xix) −*x* + 1, *y*, −*z* − 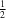; (xx) *x* + 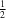, −*y* + 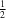, *z* − 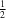; (xxi) −*x* + 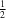, −*y* + 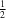, −*z* − 1; (xxii) −*x* + 1, *y*, −*z* + 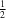; (xxiii) −*x* + 1, −*y* + 1, −*z*.]

Low-tem­per­ature data at 100 K were used for modelling and can be indexed on the basis of a smaller monoclinic cell, with *a* = 15.467 (3), *b* = 8.9627 (4) and *c* = 9.1186 (15) Å, and β = 124.1439 (15)° in the space group *C*2/*c*, with *Z* = 4. Weak additional Bragg peaks again arise from twinning (see the supporting information for more details). Here the unit-cell parameters of this smaller monoclinic cell are related to those of the rhombohedral cell by 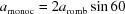
°, 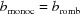 and 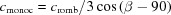. Space-group determination suggested both *C*2/*c* and acentric *Cc* symmetry; however, any attempts to refine the structure in the space group *Cc* failed to give proper *R* values (*wR*_2_ > 20%). The structure of the [Sc_2_(PO_4_)_3_]^3−^ framework, as obtained from the structure solutions in *C*2/*c*, corresponds to that proposed by Collin *et al.* (1986 ▸) for NSP, by Subramanian *et al.* (1985 ▸) for Na_3_Zr_1.5_Sc_0.5_(P_1.5_Si_1.5_O_12_) and by Hong (1976 ▸) for Na_3_Zr_2_(Si_2_PO_12_). In the final refinements, the atomic coordinates where chosen to be equivalent to these published structures. The low-tem­per­ature α-form (Fig. 9 ▸) shows one independent Sc^3+^ site on the general 8*f* position (site symmetry 1), two independent P^5+^ sites (P1 on the 8*f* and P2 on the 4*e* position with site symmetry 1 and 2, respectively) and six independent O-atom positions, all on 8*f*. Among the atomic displacement parameters, Sc again appears to vibrate almost isotropically and the two distinct P^5+^ have *U*_max_/*U*_min_ values of 2.0 and 1.8 for P1 and P2, respectively, while the O atoms again are rather anisotropic, with values between 1.6 (O4) and 4.4 (O5). The slight anisotropy of P^5+^ appears to be an intrinsic property of ASP, even at low tem­per­ature. Collin *et al.* (1986 ▸) stated that vibrational motions of the P atoms are symmetric for NSP in the monoclinic form and, thus, no positional disorder is present at low tem­per­ature.

The ScO_6_ octahedra within the first coordination sphere in the framework of the α and β phases of ASP are remarkably alike with respect to the average bond lengths and distortion indices. This also applies to the PO_4_ tetrahedra. However, the tetrahedra show a higher bond-angle variance and also higher distortion with similar volumes and average P—O bond lengths. Over the entire tem­per­ature range and in the different phases, the tetrahedra deviate most from an ideal configuration in the low-tem­per­ature α-phase. While the above-mentioned differences are com­paratively small, more pro­nounced changes occur in the arrangement of polyhedra relative to each other within and between the lantern units during the phase transition. The intra-lantern Sc—O—P angles for Sc^xvii^—O5^xviii^—P1^xvii^ are ∼153° in the β-phase and ∼159.6 (1)° in the α-phase (see Fig. 9 ▸ for symmetry codes xvii–xxiii). The Sc^xvii^—O4^xix^—P1^xix^ angles narrow to 139.5 (1)° with the Sc⋯Sc distance remaining almost constant at 4.600 (2) Å at 100 K, which is close to the value of 4.598 (1) Å at 298 K. Also, the inter-lantern Sc—O—P angles differ significantly: they cluster around 146° in the β-phase, whereas in the low-tem­per­ature α-phase, they deviate at 100 K with values of 154.1 (2), 141.5 (2) and 150.5 (2)° for the Sc^xvii^—O3^xx^—P1^xx^, Sc^xxi^—O6^xxi^—P1^xxi^ and Sc—O1—P1 angles, respectively. These rearrangements of the polyhedral interconnections directly influence the size of the bottlenecks. Two different bottlenecks can be identified and are determined by the triangles defined by the O1^xx^—O6^xx^ edge of the Sc octahedron and the O3^xxi^ and O5^xx^ corners of the opposite tetrahedron (*cf* Fig. 9 ▸). One of the bottlenecks is narrower than the other in the α-phase. The area of the smaller triangle O1^xx^—O6^xx^—O5^xx^—O1^xx^ is 5.184 Å^2^ at 100 K, which is ∼0.3 Å^2^ less than the smallest area in the β-phase, potentially hampering ion diffusion. The area of the second triangle O1^xx^—O6^xx^—O3^xxi^—O1^xx^ is 6.204 Å^2^ at 100 K.

**Figure 10:**
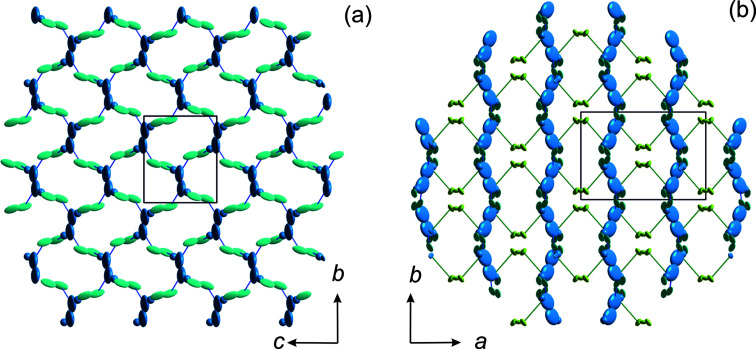
Pathways of the shortest Ag^+^⋯Ag^+^ interatomic distances in the low-tem­per­ature *C*2/*c* α-phase of Ag_3_Sc_2_(PO_4_)_3_. Note, only the Ag^+^ ions are shown for clarity (Ag1 positions in blue, Ag2 in green and Ag3 in turquoise). (*a*) A section of a single layer with *x* ∼ 0.25. (*b*) The 2+1-dimensional network viewed along [001].

Assuming full occupation, Collin *et al.* (1986 ▸) reported three different Na^+^ sites for the *C*2/*c* α-phase of NSP: two on the general 8*f* position, both half occupied, and one at the special 4*e* position (Na2 at 0, 0.1093, 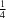). Large residual densities were found at very similar positions during structure solution of ASP at 100 K in this study. As already observed for the β- and γ-phases, the Ag-atom positions appear to be highly anisotropic and a split-site refinement was necessary. At 100 K, the regular Ag1 position, which can be com­pared to that used by Collin *et al.* (1986 ▸), has a site-occupancy factor (s.o.f.) of 0.811 (5); however, distinct residual densities were found at distances of ∼0.6 Å. To account for this, a split Ag1*A* site, with an s.o.f. of 0.105 (5), and an Ag1*B* site, with a very low s.o.f. of 0.015 (3) at a small second residual opposite to Ag1, were introduced. For the latter, only an isotropic refinement of the atomic displacement parameters was possible. The Ag2 ions reside at the 4*e* position with an s.o.f of 0.496 (2) and, thus, the site appears to be nearly fully occupied, which is similar to the findings of Collin *et al.* (1986 ▸). Both regular Ag1 and Ag2 positions exhibit small atomic displacement parameters, with *U*_eq_ ∼ 0.0123 Å, which is indicative of well-located Ag^+^ ions. A further small residual peak was detected ∼1 Å offset from the regular Ag2 position and is labelled the Ag2*A* site; thus, a small s.o.f. of 0.012 (2) at 100 K. The Ag3 site had to be split into two com­ponents, which are separated by as much as 1.3 Å, have s.o.f. values of 0.048 (3) and 0.044 (3), and are labelled Ag3*A* and Ag3*B*, respectively. These positions thus show a very low occupation with more than 90% of the split site unoccupied. This is in contrast to NSP, where the corresponding Na3 site was assumed to be 50% filled. The Ag1 and Ag2 sites in ASP are clearly filled to the same extent as the Ag3 site(s). The pathways for Ag^+^–Ag^+^ jumps have a less distinct 3D character in the low-tem­per­ature α-phase when com­pared to the β- and γ-phases. One possible pathway is along the …Ag3*B*–Ag3*A*–Ag1*B*–Ag1–Ag1*A*–Ag1*A*–Ag1–Ag1*B*–Ag3*A*–Ag3–Ag1*B*… atoms, with distances of 1.30 (3)–1.19 (7)–0.61 (7)–0.70 (3)–1.71 (5)–0.70 (3)–0.61 (7)– 1.19 (7)–1.30 (3)–2.27 (2) Å, respectively (*cf* Fig. S3 in the supporting information), which all together represent small jump distances. The atoms involved here form a crimpled two-dimensional (2D) layer, as is evident when viewing along [100] in Fig. 10 ▸(*a*). The interconnection of these layers is facilitated *via* Ag1—Ag2*A*—Ag1, with a shortest distance of 3.126 (4) Å, whereas the regular Ag1 and Ag2 sites are separated from each other by as much as 3.581 (1) Å. Thus, these are discrete layers despite the 3D linking of Ag^+^. However, the 2+1D (two- plus one-dimensional) character of the Ag^+^ network and the closing of the bottleneck suggest a low ionic conduction in the low-tem­per­ature α-phase of ASP.

**Figure 11:**
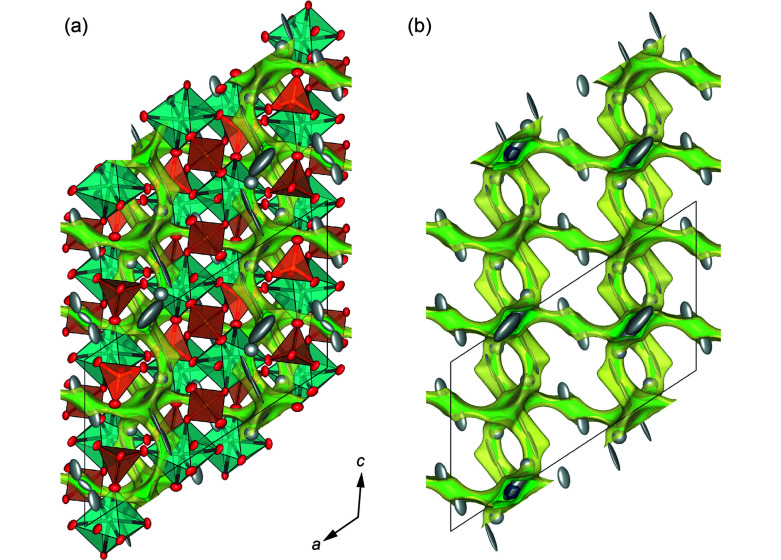
(*a*) The Ag_3_Sc_2_(PO_4_)_3_ structure in the *C*2/*c* phase at 100 K, with anisotropic atomic displacement ellipsoids at the 95% probability level com­pared with (*b*) the calculated bond-valence electron landscape map [at the 0.02 (dark blue), 0.20 (green) and 0.45 eV (yellow) level above minimum]. Note that only the experimentally obtained Ag^+^ positions are shown on the calculated BVEL map.

Inspection of the BVEL maps of the 100 K data reveals that the minimum energy in the structure (−0.4195 eV) is located at the special position 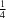, 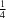, 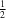. This position is unoccupied, but 1.44 Å from this position is the regular Ag1 position, with a large site occupation. The Ag1*A* position is offset a further 0.83 Å. A second minimum is located at the 4*e* position, which is coincident with the Ag2 position. Generally, the positions for Ag^+^ lie within the calculated energy minimum diffusion pathways, with an activation energy limit of 0.45 eV, as depicted in Fig. 11 ▸.

The s.o.f. values change with increasing tem­per­ature. The changes below 150 K are minor, with the Ag1 site losing some occupation, as do the Ag1*A* and Ag3 sites, and to the same extent. The s.o.f. values for the Ag2 sites remain unchanged. Things change more significantly, however, on approaching the phase-transition tem­per­ature. At 160 K, the Ag1 site occupation takes a distinct drop, whereas the Ag3 site(s) become(s) highly populated (see Table S2 in the supporting information). This can be interpreted to be due to disorder of the Ag^+^ on different sites prior to phase transition.

### 3.4. Evolution of lattice parameters with tem­per­ature   

**Figure 12:**
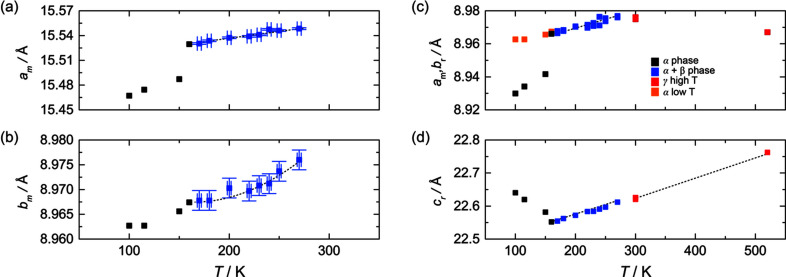
Evolution of the unit-cell parameters of Ag_3_Sc_2_(PO_4_)_3_ with changes in tem­per­ature.

The *a* and *b* unit-cell parameters are similar within the monoclinic phases and, thus, can be com­pared directly [Figs. 12 ▸(*a*) and 12 ▸(*b*)]. It is evident that upon phase transition, the *a*-lattice parameter in the β-phase increases dramatically almost linearly by about 0.04 Å. The unit-cell parameters in the α phase at 160 K better fit the trend of the β-phase; however, there is good evidence from data indexing that the *c*-lattice parameter is indeed 9.1328 (14) Å, instead of ∼22.57 Å, which would be indicative of the β-phase. The *b*-lattice parameters behave in a similar way. They increase in a slightly nonlinear way with an increase in tem­per­ature, and there is a change in slope of the data array at the α→β phase transition. The unit cells for the α- and β-phases have been subsequently transformed to the equivalent rhombohedral cells using the relationships given in the text above in order to directly com­pare the evolution of all lattice parameters as the tem­per­ature changes [Figs. 12 ▸(*c*) and 12 ▸(*d*)]. It is evident that *a*_rhomb_ does not increase as tem­per­ature increases, but rather decreases. In contrast, the *c*-lattice parameter increases linearly and perfectly fits the trend for thermal expansion of the *c*-lattice parameter in the β-phase.

## 4. Conclusion   

NaSICON-structured materials belong to the most promising group of solid electrolytes for Li-ion batteries and ‘beyond Li-ion battery’ concepts (*e.g.* Na and K) due to their superior ionic conductivities. Despite it being well known for decades, the exact phase behaviour is poorly understood. One of the reasons for this is the absence of suitable single crystals that could enable an in-depth investigation of the structure, site preference and phase behaviour as a function of tem­per­ature. Moreover, the low scattering contrast of Na and, in particular, Li makes their analysis particularly difficult.

In this study, we grew NSP single crystals by the flux method and subsequently chemically exchanged Na^+^ by Ag^+^ in order to take advantage of the higher scattering contrast of Ag^+^. This Na^+^–Ag^+^ exchange is fast and associated with an increase of lattice parameters and unit-cell volume. The main focus of this study was the full structural characterization of Ag_3_Sc_2_(PO_4_)_3_ throughout the proposed phase-transition sequence and to evaluate its tem­per­ature dependence.

We found that the NaSICON-type com­pound shows two phase transitions from a low-tem­per­ature monoclinic α-phase to a monoclinic β-phase at about 170 K and to a rhombohedral γ-phase at about 280 K. The framework of [Sc_2_(PO_4_)_3_]^3−^ is rigid and does not change significantly with tem­per­ature and change of symmetry. Major differences are found in the distribution of Ag^+^, especially during the transition from the β-phase to the γ-phase, suggesting that this transition is actually an order–disorder transition. Furthermore, the transition from the α- to the β-phase is associated with major changes in Ag^+^-site occupation. Distinct reorientations within the interconnections of polyhedra also occur. Therefore, the main driving force of this transition may also be due to an order–disorder of Ag^+^, which induces some displacive structural adjustments. Although there is highly anisotropic atomic displacement and splitting of the Ag positions in places, the experimentally refined total amount of Ag^+^ at different tem­per­atures and in the various structural states is in the range 3.02–3.05 apfu. It is acknowledged that the small observed scatter might be a real effect and could reflect a very small deficit in the phospho­rous content.

The sensitivity of the phase behaviour on the ordering of these ions suggests that small com­positional changes can have a great impact on the phase behaviour and, therefore, also on the ionic conductivity of NaSICON-structured materials.

## Supplementary Material

Crystal structure: contains datablock(s) global, 100K, 200K, 300K, 520K. DOI: 10.1107/S2052520620014870/rm5035sup1.cif

Structure factors: contains datablock(s) 100K. DOI: 10.1107/S2052520620014870/rm5035100Ksup2.hkl

Structure factors: contains datablock(s) 200K. DOI: 10.1107/S2052520620014870/rm5035200Ksup3.hkl

Structure factors: contains datablock(s) 300K. DOI: 10.1107/S2052520620014870/rm5035300Ksup4.hkl

Structure factors: contains datablock(s) 520K. DOI: 10.1107/S2052520620014870/rm5035520Ksup5.hkl

Additional notes, tables and figures. DOI: 10.1107/S2052520620014870/rm5035sup6.pdf

CCDC references: 2043360, 2043361, 2043362, 2043363

## Figures and Tables

**Table 1:** Experimental details Experiments were carried out with Mo *K*α radiation using a Bruker SMART APEX diffractometer. Absorption was corrected for by multi-scan methods (*APEX2*; Bruker, 2012 ▸).

	*T* = 100 K	*T* = 200 K	*T* = 300 K	*T* = 520 K
Crystal data				
Chemical formula	Ag_3.06_Sc_2_(PO_4_)_3_ †	Ag_3.03_Sc_2_(PO_4_)_3_ †	Ag_3.05_Sc_2_(PO_4_)_3_ †	Ag_3.02_Sc_2_(PO_4_)_3_ †
*M*_r_	704.90	701.67	703.49	700.59
Crystal system, space group	Monoclinic, *C*2/*c*	Monoclinic, *C*2/*c*	Trigonal, *R* 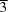 *c*:*H*	Trigonal, *R* 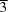 *c*:*H*
Temperature (K)	100	200	295	520
*a*, *b*, *c* (Å)	15.467 (3), 8.9627 (14), 9.1186 (15)	15.5374 (2), 8.9703 (1), 22.5718 (3)	8.9756 (4), 8.9756 (4), 22.6248 (10)	8.9671 (8), 8.9671 (8), 22.7622 (19)
α, β, γ (°)	90, 124.1439 (15), 90	90, 89.9984 (4), 90	90, 90, 120	90, 90, 120
*V* (Å^3^)	1046.2 (3)	3145.95 (7)	1578.49 (16)	1585.1 (3)
*Z*	4	12	6	6
μ (mm^−1^)	7.38	7.15	7.31	7.24
Crystal size (mm)	0.14 × 0.14 × 0.09	0.14 × 0.14 × 0.09	0.14 × 0.14 × 0.09	0.12 × 0.12 × 0.08

Data collection				
*T*_min_, *T*_max_	0.38, 0.52	0.51, 0.65	0.38, 0.52	0.417, 0.581
No. of measured, independent and observed [*I* > 2σ(*I*)] reflections	5927, 1407, 1355	50538, 6307, 2271	7548, 496, 472	4628, 924, 700
*R*_int_	0.032	0.038	0.027	0.038
(sin θ/λ)_max_ (Å^−1^)	0.706	0.769	0.701	0.873

Refinement				
*R*[*F* ^2^ > 2σ(*F* ^2^)], *wR*(*F* ^2^), *S*	0.035, 0.087, 1.22	0.032, 0.107, 1.04	0.018, 0.044, 1.11	0.033, 0.071, 1.07
No. of reflections	1407	6307	496	924
No. of parameters	140	380	60	60
No. of restraints	12	42	0	0
Δρ_max_, Δρ_min_ (e Å^−3^)	1.16, −1.19	1.08, −0.92	0.39, −0.36	0.78, −0.64

Computer programs: *APEX2* (Bruker, 2012 ▸), *APEX3* (Bruker, 2015 ▸), *SHELXL2014* (Sheldrick, 2015*b* ▸), *SHELXT* (Sheldrick, 2015*a* ▸), *ORTEP for Windows* (Farrugia, 2012 ▸) and *WinGX* (Farrugia, 2012 ▸).

† Unconstrained refinements were used so the different Ag content results in uncertainity in determining the Ag content from the single crystal data. Over the whole *T*-range it is in between 3.02 and 3.06 atoms per formula unit.

**Table 2:** Selected geometric parameters (Å) for Ag_3_Sc_2_(PO_4_)_3_ at different tem­per­atures

***T* = 100 K**			
Sc1—O4^i^	2.035 (5)	P2—O2^iv^	1.513 (4)
Sc1—O2^i^	2.038 (4)	P2—O6	1.554 (4)
Sc1—O3	2.071 (4)	Ag1⋯Ag1*B*	0.61 (7)
Sc1—O5^ii^	2.089 (4)	Ag1⋯Ag1*A*	0.70 (3)
Sc1—O1	2.141 (4)	Ag1*A*⋯Ag1*A* ^iii^	1.71 (5)
Sc1—O6	2.150 (4)	Ag1*A*⋯Ag3*B* ^v^	2.27 (2)
P1—O5^iii^	1.514 (4)	Ag1*B*⋯Ag3*A*	1.19 (7)
P1—O3	1.527 (4)	Ag2⋯Ag2*A*	0.98 (6)
P1—O4	1.539 (5)	Ag3*A*⋯Ag3*B*	1.30 (3)
P1—O1^iv^	1.554 (4)		

***T* = 200 K**			
Sc1—O5	2.045 (4)	P4—O13	1.522 (4)
Sc1—O13	2.049 (4)	P4—O14	1.547 (4)
Sc1—O8^vi^	2.052 (4)	P5—O17	1.524 (4)
Sc1—O9	2.124 (4)	P5—O16	1.525 (4)
Sc1—O15	2.133 (3)	P5—O15	1.535 (4)
Sc1—O1	2.136 (4)	P5—O18	1.537 (4)
Sc2—O2^i^	2.044 (3)	Ag1*A*⋯Ag1*B*	0.628 (6)
Sc2—O16^vii^	2.047 (4)	Ag1*A*⋯Ag6*B* ^x^	2.800 (15)
Sc2—O10^viii^	2.051 (4)	Ag1*B*⋯Ag7*C*	2.748 (18)
Sc2—O3^ix^	2.126 (4)	Ag1*B*⋯Ag7*B*	2.977 (14)
Sc2—O12^x^	2.132 (3)	Ag2*A*⋯Ag2*B*	0.620 (5)
Sc2—O18	2.135 (3)	Ag2*A*⋯Ag6*C*	2.906 (14)
Sc3—O11^x^	2.047 (4)	Ag2*B*⋯Ag7*B* ^viii^	2.632 (17)
Sc3—O4	2.048 (4)	Ag2*B*⋯Ag7*A* ^viii^	2.837 (15)
Sc3—O17^xi^	2.052 (4)	Ag3*A*⋯Ag3*B*	0.607 (6)
Sc3—O14^xii^	2.124 (3)	Ag3*A*⋯Ag6*A*	2.865 (10)
Sc3—O6^vi^	2.127 (4)	Ag3*B*⋯Ag7*A*	2.654 (11)
Sc3—O7^x^	2.129 (4)	Ag3*B*⋯Ag7*C* ^viii^	2.977 (10)
P1—O4	1.527 (5)	Ag4⋯Ag4^vi^	0.564 (13)
P1—O2	1.534 (4)	Ag4⋯Ag6*A*	2.812 (11)
P1—O1	1.540 (4)	Ag5*A*⋯Ag5*B*	0.628 (4)
P1—O3	1.543 (4)	Ag5*A*⋯Ag6*B* ^xiii^	2.862 (14)
P2—O8	1.525 (4)	Ag5*B*⋯Ag6*C*	2.822 (17)
P2—O5	1.533 (4)	Ag6*A*⋯Ag6*C* ^xi^	0.897 (9)
P2—O7	1.536 (4)	Ag6*A*⋯Ag6*B* ^xi^	0.981 (7)
P2—O6	1.541 (4)	Ag6*B*⋯Ag6*C*	0.899 (7)
P3—O10	1.526 (4)	Ag7*A*⋯Ag7*B*	0.695 (9)
P3—O11	1.536 (4)	Ag7*A*⋯Ag7*C* ^viii^	0.704 (9)
P3—O9	1.538 (4)	Ag7*B*⋯Ag7*C*	0.674 (9)
P3—O12	1.541 (3)		

***T* = 300 K**			
Sc1—O1^xi^	2.0505 (12)	Ag11⋯Ag11^xv^	0.668 (3)
Sc1—O2	2.1324 (11)	Ag11⋯Ag21	1.35 (3)
P1—O1^xi^	1.5261 (12)	Ag21⋯Ag22	1.18 (3)
P1—O2^xiv^	1.5398 (11)	Ag22⋯Ag2	0.501 (12)

***T* = 520 K**			
Sc1—O1^xi^	2.0542 (14)	Ag11⋯Ag11^xv^	0.748 (3)
Sc1—O2	2.1334 (13)	Ag11⋯Ag21	1.310 (19)
P1—O1^xi^	1.5215 (14)	Ag21⋯Ag22	1.03 (2)
P1—O2^xiv^	1.5360 (14)	Ag22⋯Ag2	0.623 (11)

Symmetry codes: (i) −*x* + 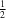, −*y* + 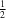, −*z*; (ii) −*x*, *y*, −*z* + 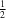; (iii) −*x* + 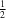, −*y* + 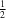, −*z* + 1; (iv) −*x* + 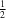, *y* + 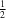, −*z* + 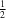; (v) *x*, −*y*, *z* + 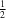; (vi) −*x* + 1, *y*, −*z* + 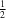; (vii) −*x* + 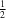, −*y* + 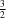, −*z*; (viii) −*x* + 1, −*y* + 1, −*z*; (ix) *x* − 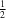, *y* + 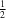, *z*; (x) *x* − 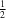, *y* − 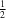, *z*; (xi) *x*, *y* − 1, *z*; (xii) −*x* + 1, *y* − 1, −*z* + 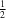; (xiii) −*x* + 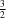, *y* − 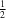, −*z* + 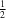; (xiv) −*x* + 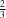, −*y* + 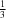, −*z* + 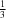; (xv) *x* − *y*, *x*, −*z*.
