# Supplementary material for: Study on the structural phase transitions in NaSICON-type compounds using Ag3Sc2(PO4)3 as a model system
Source: Acta Crystallogr B Struct Sci Cryst Eng Mater. 2020 Dec 24;77(Pt 1):10–22. doi: 10.1107/S2052520620014870 (PMC7941282; doi:10.1107/S2052520620014870)
Supplement: Supplementary file 6 [file b-77-00010-sup6.pdf]

STRUCTURAL SCIENCE
CRYSTAL ENGINEERING
MATERIALS

Volume 77 (2021)

Supporting information for article:

Study on the structural phase transitions in NaSICON-type compounds using Ag3Sc2(PO4)3 as a model system

Günther J. Redhammer, Gerold Tippelt, Quirin Stahl, Artur Benisek and Daniel Rettenwander

Notes on the indexing of the β - phase of $Ag_3Sc_2(PO_4)_3$

From Figure 7b in the main text it is evident that weaker reflections in addition to the strong ones appear. Precession images of that type have already been presented by Collin *et al.* (1986) in a very similar way for $Na_3Sc_2(PO4)_3$. Indexing of all these observed intensity data is possible on basis of a hexagonal unit cell with lattice parameters *a* = *b* = 18.060(8) Å and *c* = 22.661(11) Å with space group symmetry $P\overline{3}c1$. However, no adequate structure solution was possible with this data reduction model, in agreement with previous findings of *e.g.* Collin *et al.* (1986), (Ladenstein *et al.*, 2020). Best models stuck around $wR_2 \sim 40$ %. As the superstructure reflections can be described by $2a$, $2b$, c of the parent cell of the γ phase, it was tested to describe the satellites using an incommensurate modulation. Only two-dimensional modulation can describe all reflections within $R\overline{3}c(p00,0p0)0m$ with q_1 ~0.495 0 0 and q_2 (0 0.498 0), but we could not resolve the incommensurate structure, while the parent structure was refined to low R-values on basis of the rhombohedral structure of the γ-phase. As the superstructure could not be described, we dismissed this model.

Assuming modulation, a more plausible structural model was derived using a small monoclinic unit cell with *a* = 15.628(6) Å, *b* = 9.016(4) Å, *c* = 9.170(4) Å and β= 124.619(5)°, space group symmetry *C*2/*c*. Full indexing of diffraction data is possible using an commensurable modulation along **c** – direction (with q_1 ~ 1.0, 0, 0.333) but only in combination with a tri-twinning of the crystal. For this data reduction, the average structure could be refined down to low R-values, however, the modulated structure could not be resolved. So finally, we decided to describe the β - phase in the cell given in the text with *a* = 15.5374(2) Å, *b* = 8.9703(1) Å, *c* = 22.5718(3) Å and β= 89.998(4)° at 200K and assuming a tri-twinning. With this model it is possible to describe about 99.7 % of all observed Bragg peaks and reliable structure models are obtained.

Notes on the indexing of the low temperature α - phase

In detailed examination of the simulated precession images in Figure 7c, weak additional peaks are visible, which also arise from the twinning of the crystals in due course of the phase transitions. In data reduction, somewhat more than 94 % of the Bragg peaks can be indexed on basis of the cell of the α - phase with *a* = 15.467(3) Å, *b* = 8.9627(4) Å, *c* = 9.1186(15) Å, and β = 124.1439(15) °. The remaining reflections (except < 0.4 %) can be indexed with the same cell using a twin matrix given below and using in the refinement

$$\text{Twin matrix } \beta \text{ - phase} = \begin{pmatrix} 0.5 & -1.5 & 0 \\ -0.5 & -0.5 & 0 \\ 0 & 0 & -1 \end{pmatrix}$$

$$\text{Twin matrix } \alpha \text{ - phase} = \begin{pmatrix} -0.5 & -1.5 & 0 \\ 0.5 & -0.5 & 0 \\ 0.5 & 0.5 & 1 \end{pmatrix}$$

For the α - phase, no evidence for an (in)commensurate modulation was found.

Table S1 Lattice parameters of $Ag_3Sc_2(PO_4)_3$, obtained from $Na_3Sc_2(PO_4)_3$ powder during different immersion times in a 1M $AgNO_3$ solution.

Immersion time (min)	a (Å)	esd a	c (Å)	esd c
1	8.9708	0.0003	22.6129	0.0006
3	8.9721	0.0003	22.6154	0.0004
6	8.9724	0.0002	22.6158	0.0004
9	8.9735	0.0003	22.6174	0.0003
14	8.9741	0.0002	22.6183	0.0003
15	8.9743	0.0003	22.6187	0.0003
15	8.9746	0.0003	22.6181	0.0004
30	8.9754	0.0003	22.6200	0.0004
60	8.9759	0.0003	22.6211	0.0004
120	8.9769	0.0003	22.6232	0.0003
180	8.9778	0.0003	22.6232	0.0004
180	8.9773	0.0002	22.6234	0.0003
240	8.9780	0.0002	22.6242	0.0005
420	8.9784	0.0002	22.6247	0.0003
600	8.9795	0.0002	22.6257	0.0004
1380	8.9793	0.0003	22.6258	0.0003
3900	8.9798	0.0003	22.6254	0.0004
6840	8.9796	0.0002	22.6261	0.0004
6840	8.9798	0.0002	22.6263	0.0004

Table S2 Refined site occupation factors (s.o.f.) for Ag^+ at different temperatures in three different modifications (a), (b) and (c).

(a) $Ag_3Sc_2(PO_4)_3$ in the $R\bar{3}c$ γ - phase.

Sample ID	T (K)	s.o.f. Ag11	s.o.f. Ag21	s.o.f. Ag22	s.o.f. Ag2
Ag-NSP_F6_2[(a)]	300 K	0.1688(8)	0.0085(10)	0.140(9)	0.190(9)
F6_QS_300K	300 K	0.1693(7)	0.0075(10)	0.136(9)	0.192(9)
F6_QS_520 K[(b)]	520 K	0.1692(6)	0.0136(12)	0.142(7)	0.179(7)
Ag-NSP_F7_1[(c)]	300 K	0.1694(6)	0.0083(9)	0.135(7)	0.193(7)
Ag-NSP_F7_2[(c)]	300 K	0.1690(6)	0.0082(10)	0.140(9)	0.188(9)
Ag-NSP_F7_T300_1[(d)]	300 K	0.1693(6)	0.0079(9)	0.148(9)	0.179(9)
Ag-NSP_F7_T300_2[(d)]	300 K	0.1694(6)	0.0091(9)	0.132(7)	0.195(7)

(a) corresponds to the structure refinement at 300 K discussed in the text ; (b) corresponds to the 520 K structure discussed in the text, for a crystal from the same batch as Ag-NSP-F6_2; (c) crystals selected from a different batch to check for any between-batch differences (d) refinement of crystals from batch F7, but annealed at 600 K for a period of 4 days to check for low temperature Ag^+ ordering.

(b) $Ag_3Sc_2(PO_4)_3$ in the $C2/c$ β - phase, split positions are added together.

T (K)	s.o.f. Ag1	s.o.f. Ag2	s.o.f. Ag3	s.o.f. Ag4	s.o.f. Ag5	s.o.f. Ag6	s.o.f. Ag7
180 K	0.689	0.660	0.663	0.323	0.679	1.024	0.510
200 K	0.685	0.659	0.665	0.321	0.678	1.029	0.512
250 K	0.676	0.662	0.662	0.325	0.671	1.027	0.509
270 K	0.673	0.659	0.662	0.329	0.669	1.022	0.507

(c) $Ag_3Sc_2(PO_4)_3$ in the $C2/c$ α - phase, split positions are added together.

T (K)	s.o.f. Ag1	s.o.f. Ag2	s.o.f. Ag3
100	0.931(5)	0.508(2)	0.090(2)
115	0.908(6)	0.511(2)	0.101(2)
150	0.874(5)	0.520(2)	0.132(2)
160	0.507(7)	0.339(5)	0.676(5)

Table S3 Selected bond lengths and distortional parameters for $Ag_3Sc_2(PO_4)_3$ in the α- , β- and γ-phase as a function of temperature. Interatomic distances are given in Å, and bond angles in °.

(a) $R\bar{3}c$ - phase

	$Ag_3Sc_2(PO_4)_3$		$Na_3Sc_2(PO_4)_3$
T (K)	300K	520K	502 K
Sc1-O1	2.0504(18)	2.055(3)	2.050(3)
Sc1-O2	2.1324(11)	2.1333(13)	2.1123(16)
Sc1-O2	2.1324(11)	2.1333(13)	2.1123(16)
Sc1-O1	2.0504(18)	2.055(3)	2.050(3)
Sc1-O2	2.1324(11)	2.1333(14)	2.1123(16)
Sc1-O1	2.0504(18)	2.055(2)	2.050(3)
<Sc1-O>	2.0914	2.094	2.0809
Volume (Å^3)	12.11	12.18	11.91
Dist.Ind.	0.0196	0.01877	0.01507
OQE	1.005	1.004	1.0058
BAV	16.51	12.98	19.82
ECON	5.92	5.92	5.95
P1-O1	1.5263(15)	1.5212(18)	1.526(3)
P1-O1	1.5263(15)	1.5212(18)	1.526(3)
P1-O2	1.5398(11)	1.5360(13)	1.5210(14)
P1-O2	1.5398(11)	1.5360(13)	1.5210(13)
<P1-O>	1.533	1.529	1.523
Volume (Å^3)	1.85	1.83	1.81
Dist.Ind	0.00442	0.00485	0.00155
TQE	1.0005	1.0005	1.0011
BAV	2.13	2.14	4.45
ECON	4.00	4.00	4.00
Sc1-O1-P1	153.22(10)	153.17(10)	151.32(12)
Sc1-O1-P1	146.42(8)	147.49(9)	147.43(11)
Bottleneck Areas			
T2 (Å^2)	5.952	6.071	5.598
T1 (Å^2)	5.495	5.555	5.384

$^{\&}$ (Ladenstein *et al.*, 2020). Vol. = Polyhedral volume. Dist.Ind. = Distortion index as defined by (Baur, 1974) with $D = \frac{1}{n}\sum_{i=1}^{n}\frac{|l_i - l_{av}|}{l_{av}}$, where l_i is the distance from the central atom to the ith coordinating atom. l_{av} is the average bond length. OQE = octahedral quadratic elongation $\langle\lambda\rangle$. TQO = tetrahedral quadratic elongation $\langle\lambda\rangle$ as defined by (Robinson *et al.*, 1971) with $\langle\lambda\rangle = \frac{1}{n}\sum_{i=1}^{n}\left(\frac{l_i}{l_0}\right)^2$ where l_0 is the centre-to-vertex distance of a regular polyhedron with the same volume. BAV = bond angle variance σ^2 as defined by (Robinson *et al.*, 1971) with $\sigma^2 = \frac{1}{m-1}\sum_{i=1}^{n}(\phi_i - \phi_0)^2$ where m is the number of bond angles. ϕ_i is the ith bond angle and ϕ_0 is the ideal bond angle of a regular polyhedron. ECON = effective coordination number. All calculations were done using VESTA (Momma & Izumi, 2011).

(b) $Ag_3Sc_2(PO_4)_3$ in the *C*2/*c* β - phase

	$Ag_3Sc_2(PO_4)_3$				$Na_3Sc_2(PO_4)_3$
T (K)	180	200	250	270	300
Sc1-O13	2.044(4)	2.049(4)	2.050(5)	2.059(6)	2.055(3)
Sc1-O9	2.127(4)	2.125(4)	2.125(5)	2.123(6)	2.121(3)
Sc1-O5	2.042(4)	2.045(5)	2.039(5)	2.053(7)	2.046(3)
Sc1-O15	2.135(4)	2.133(4)	2.133(5)	2.139(5)	2.119(3)
Sc1-O8	2.050(4)	2.052(5)	2.053(5)	2.061(7)	2.060(4)
Sc1-O1	2.129(4)	2.136(4)	2.134(5)	2.136(6)	2.114(3)
<Sc1-O>	2.088	2.090	2.089	2.095	2.086
Volume (Å^3)	12.048	12.081	12.070	12.187	11.962
Dist.Ind	0.02035	0.01972	0.0199	0.0180	0.0154
OQE	1.0054	1.0052	1.0050	1.0045	1.0079
BAV	17.56	17.14	16.48	14.93	26.93
ECON	5.91	5.91	5.91	5.93	5.95
Sc2-O10	2.055(4)	2.052(5)	2.052(5)	2.067(7)	2.056(3)
Sc2-O18	2.134(4)	2.134(4)	2.138(5)	2.134(5)	2.120(3)
Sc2-O12	2.121(4)	2.132(4)	2.133(5)	2.129(7)	2.117(3)
Sc2-O2	2.044(4)	2.043(5)	2.044(5)	2.047(7)	2.050(3)
Sc2-O16	2.049(4)	2.048(4)	2.053(5)	2.055(6)	2.052(3)
Sc2-O3	2.128(4)	2.126(4)	2.135(5)	2.132(5)	2.120(3)
<Sc2-O>	2.089	2.089	2.092	2.094	2.086
Volume (Å^3)	12.059	12.064	12.127	12.149	11.961
Dist.Ind	0.01866	0.01985	0.02044	0.018	0.016
OQE	1.0053	1.0055	1.0052	1.0053	1.008
BAV	17.39	18.06	17.12	17.47	27.543
ECON	5.92	5.91	5.90	5.92	5.944
Sc3-O4	2.044(4)	2.048(5)	2.063(5)	2.044(6)	2.057(4)
Sc3-O6	2.120(4)	2.127(4)	2.128(5)	2.122(6)	2.114(2)
Sc3-O14	2.130(4)	2.124(4)	2.131(5)	2.137(5)	2.121(3)
Sc3-O17	2.053(4)	2.052(4)	2.058(5)	2.060(6)	2.054(3)
Sc3-O11	2.045(4)	2.047(5)	2.051(5)	2.046(6)	2.049(3)
Sc3-O7	2.133(4)	2.129(4)	2.128(5)	2.124(5)	2.120(3)
Sc3-O	2.087	2.088	2.093	2.088	2.086
Volume (Å^3)	12.036	12.049	12.145	12.057	11.963
Dist.Ind	0.0192	0.01867	0.0172	0.01893	0.01563
OQE	1.0054	1.0051	1.0048	1.0051	1.0079
BAV	17.89	17.01	16.14	16.86	26.88
ECON	5.92	5.92	5.93	5.92	5.95
P1-O4	1.528(4)	1.527(5)	1.517(5)	1.527(7)	1.508(4)
P1-O3	1.541(4)	1.543(4)	1.537(5)	1.544(6)	1.530(3)
P1-O1	1.537(3)	1.540(4)	1.533(5)	1.537(6)	1.534(2)

P1-O2	1.531(4)	1.534(5)	1.532(5)	1.533(7)	1.537(3)
<P1-O>	1.534	1.536	1.530	1.535	1.527
Volume (Å^3)	1.851	1.859	1.835	1.855	1.824
Dist.Ind	0.00316	0.00361	0.0043	0.0035	0.00636
TQE	1.0007	1.0006	1.0007	1.0007	1.0014
BAV	2.57	2.54	2.82	2.88	5.47
ECON	4.00	4.00	4.00	4.00	3.99
P2-O5	1.533(4)	1.532(5)	1.533(5)	1.525(7)	1.535(3)
P2-O8	1.525(4)	1.526(5)	1.523(5)	1.516(7)	1.509(4)
P2-O7	1.534(4)	1.536(4)	1.538(5)	1.544(6)	1.531(3)
P2-O6	1.543(3)	1.541(4)	1.547(5)	1.550(6)	1.534(2)
<P2-O>	1.534	1.534	1.535	1.534	1.527
Volume (Å^3)	1.850	1.850	1.855	1.850	1.824
Dist.Ind	0.00319	0.00295	0.00462	0.00875	0.0061
TQE	1.0006	1.0006	1.0006	1.0007	1.0014
BAV	2.18	2.31	2.13	2.33	5.53
ECON	4.00	4.00	4.00	3.99	3.99
P3-O9	1.535(4)	1.537(4)	1.541(5)	1.538(6)	1.530(3)
P3-O12	1.541(3)	1.541(4)	1.535(5)	1.545(6)	1.535(2)
P3-O10	1.520(4)	1.526(5)	1.530(5)	1.511(7)	1.512(3)
P3-O11	1.531(4)	1.536(5)	1.524(5)	1.536(7)	1.533(3)
<P3-O>	1.532	1.535	1.533	1.533	1.528
Volume (Å^3)	1.844	1.854	1.846	1.846	1.826
Dist.Ind	0.00419	0.00292	0.00355	0.007	0.00498
TQE	1.0005	1.0006	1.0006	1.0007	1.0014
BAV	1.98	2.54	2.39	2.36	5.40
ECON	4.00	4.00	4.00	3.99	4.00
P4-O14	1.543(4)	1.547(4)	1.541(5)	1.538(6)	1.531(3)
P4-O13	1.534(4)	1.522(5)	1.525(5)	1.517(7)	1.507(3)
P4-O13	1.534(4)	1.522(5)	1.525(5)	1.517(7)	1.507(3)
P4-O14	1.543(4)	1.547(4)	1.541(5)	1.538(6)	1.531(3)
<P4-O>	1.539	1.534	1.533	1.527	1.519
Volume (Å^3)	1.867	1.852	1.847	1.827	1.796
Dist.Ind	0.00285	0.00822	0.0053	0.00696	0.00781
TQE	1.0007	1.0005	1.0006	1.0007	1.0007
BAV	2.66	1.69	2.22	2.79	2.40
ECON	4.00	3.99	4.00	3.99	3.99
P5-O15	1.539(4)	1.536(4)	1.544(6)	1.534(6)	1.531(3)
P5-O18	1.535(4)	1.537(4)	1.534(6)	1.543(6)	1.530(3)
P5-O17	1.528(4)	1.524(5)	1.521(5)	1.517(7)	1.510(3)
P5-O16	1.530(4)	1.525(5)	1.525(5)	1.521(7)	1.511(3)
<P5-O>	1.533	1.531	1.531	1.529	1.521

Volume (Å^3)	1.847	1.838	1.840	1.832	1.803
Dist.Ind	0.00267	0.00396	0.0053	0.00634	0.00668
TQE	1.0007	1.0005	1.0006	1.0006	1.0007
BAV	2.77	2.10	2.45	2.34	2.58
ECON	4.00	4.00	4.00	3.99	3.99
Sc1-Sc1	4.5910	4.5916	4.5950	4.6020	4.5029
Sc2-Sc3	4.5897	4.5936	4.5960	4.5930	4.5044
		intra – lantern angles			
Sc1-O5-P2	153.4(1)	153.3(1)	154.0(1)	153.7(1)	151.5(1)
Sc1-O8-P2	153.0(1)	152.9(1)	153.0(1)	153.6(1)	152.1(1)
Sc1-O13-P4	152.8(1)	153.8(1)	153.2(1)	153.2(1)	153.4(1)
Sc2-O16-P5	153.0(1)	153.4(1)	153.2(1)	153.3(1)	153.8(1)
Sc3-O17-P5	153.7(1)	153.6(1)	153.9(1)	153.0(1)	153.2(1)
Sc3-O11-P3	153.6(1)	153.1(1)	154.0(1)	153.6(1)	151.2(1)
Sc2-O10-P3	153.4(1)	153.0(1)	152.6(1)	152.7(1)	152.5(1)
Sc2-O2-P1	153.2(1)	152.9(1)	153.5(1)	152.9(1)	150.4(1)
Sc3-O4-P1	153.1(1)	153.2(1)	153.2(1)	153.2(1)	153.1(1)
		inter – lantern angles			
Sc1-O15-P5	145.7(1)	146.4(1)	146.0(1)	146.3(1)	144.3(1)
Sc1-O9-P3	146.4(1)	146.4(1)	146.3(1)	147.0(1)	144.6(1)
Sc1-O1-P1	146.3(1)	145.8(1)	146.5(1)	146.3(1)	144.6(1)
Sc2-O12-P3	146.3(1)	145.7(1)	146.4(1)	146.0(1)	144.5(1)
Sc2-O3-P1	146.9(1)	146.0(1)	146.3(1)	146.4(1)	144.6(1)
Sc2-O18-P5	146.1(1)	145.8(1)	146.1(1)	145.8(1)	144.3(1)
Sc3-O6-P2	146.0(1)	145.8(1)	145.7(1)	146.5(1)	144.4(1)
Sc3-O7-P2	146.7(1)	146.8(1)	146.9(1)	146.5(1)	144.8(1)
Sc3-O14-P4	145.8(1)	145.9(1)	146.2(1)	146.1(1)	144.0(1)
		Bottleneck involving Sc1-octahedron			
T1 (Å^2)	5.486	5.479	5.483	5.503	5.321
T2(Å^2)	5.918	5.920	5.926	5.948	5.670
		Bottleneck involving Sc2-octahedron			
T3 (Å^2)	5.468	5.454	5.506	5.480	5.259
T4 (Å^2)	5.907	5.886	5.925	5.931	5.671
		Bottleneck involving Sc3-octahedron			
T5 (Å^2)	5.478	5.472	5.514	5.499	5.251
T6 (Å^2)	5.923	5.909	5.950	5.938	5.664

Notation as in (a)

(c) $Ag_3Sc_2(PO_4)_3$ in the *C*2/*c* α - phase

T (K)	100	115	150	160
Sc1-O3	2.071(4)	2.136(5)	2.072(4)	2.128(4)
Sc1-O1	2.141(5)	2.074(4)	2.137(4)	2.130(4)
Sc1-O4	2.035(6)	2.034(6)	2.040(6)	2.047(7)
Sc1-O6	2.150(6)	2.152(6)	2.151(5)	2.132(6)
Sc1-O2	2.038(5)	2.037(5)	2.085(5)	2.043(5)
Sc1-O5	2.088(5)	2.091(5)	2.040(4)	2.046(5)
<Sc1-O>	2.087	2.087	2.087	2.088
Volume (Å^3)	12.032	12.035	12.033	12.042
Dist.Ind	0.01876	0.01875	0.01805	0.02034
OQE	1.0056	1.0055	1.0056	1.0054
BAV	17.99	17.77	18.15	17.60
ECON	5.89	5.89	5.90	5.91
P1-O3	1.526(6)	1.524(6)	1.529(5)	1.538(6)
P1-O1	1.554(5)	1.559(5)	1.557(4)	1.540(4)
P1-O4	1.540(6)	1.537(6)	1.531(6)	1.528(7)
P1-O5	1.515(4)	1.512(4)	1.513(4)	1.526(4)
<P1-O>	1.534	1.533	1.533	1.533
Volume (Å^3)	1.849	1.847	1.845	1.847
Dist.Ind	0.0087	0.00974	0.00787	0.00384
TQE	1.001	1.001	1.001	1.0006
BAV	3.50	3.54	3.63	2.26
ECON	3.99	3.98	3.99	4.00
P2-O2	1.513(5)	1.513(4)	1.508(4)	1.532(5)
P2-O6	1.553(6)	1.550(6)	1.554(5)	1.538(6)
P2-O2	1.513(5)	1.513(4)	1.508(4)	1.532(5)
P2-O6	1.553(6)	1.550(6)	1.554(5)	1.538(6)
<P2-O>	1.533	1.532	1.531	1.535
Volume (Å^3)	1.847	1.843	1.838	1.853
Dist.Ind	0.01316	0.01203	0.01508	0.002
TQE	1.0009	1.0009	1.001	1.0005
BAV	3.61	3.46	3.70	2.07
ECON	3.98	3.98	3.97	4.00
Sc-Sc	4.6000(14)	4.5969(13)	4.5879(13)	4.5890(13)
		intra - lantern angles		
Sc-O5-P1	139.5(3)	139.5(3)	140.0(3)	153.3(3)
Sc-O4-P1	159.6(3)	159.6(3)	159.6(3)	153.3(3)
Sc-O2-P2	154.1(3)	154.2(3)	154.0(3)	153.3(3)
		inter - lantern angles		
Sc1-O3-P1	154.0(3)	154.0(3)	154.0(3)	145.9(3)
Sc1-O6-P1	141.5(3)	141.6(3)	141.8(3)	145.9(2)

Sc1-O1-P1	150.5(3)	150.5(3)	150.5(3)	145.9(3)
		Bottlenecks		
T1	5.184	5.181	5.191	5.46
T2	6.204	6.194	6.169	5.888

Notation as in Table S3a

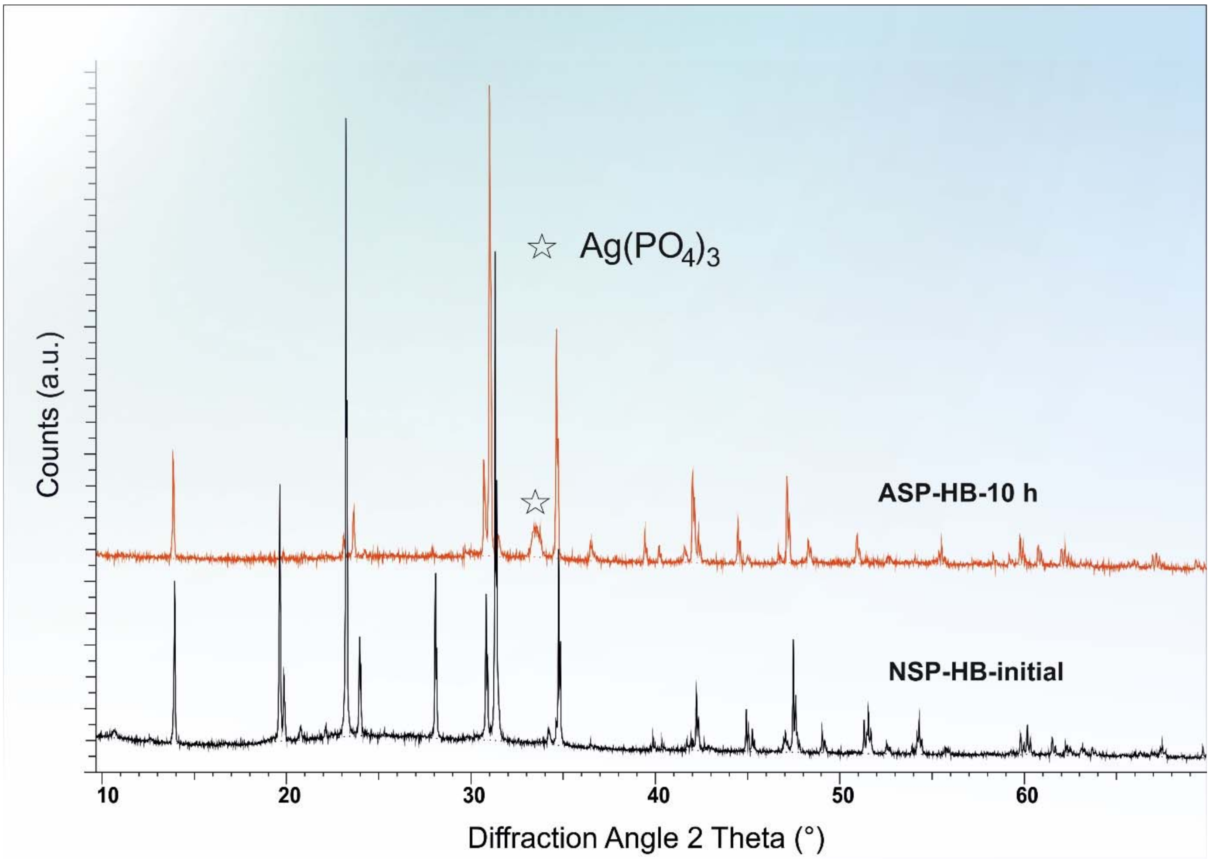

Figure S1 Comparison of the powder X-ray diffraction pattern of untreated initial $Na_3Sc_2(PO_4)_3$ polycrystalline material and that obtained after 10 h immersion in a 1M $AgNO_3$ aqueous solution. The star indicates the $Ag(PO_4)_3$ impurity (~ 4 wt % from Rietveld refinement).

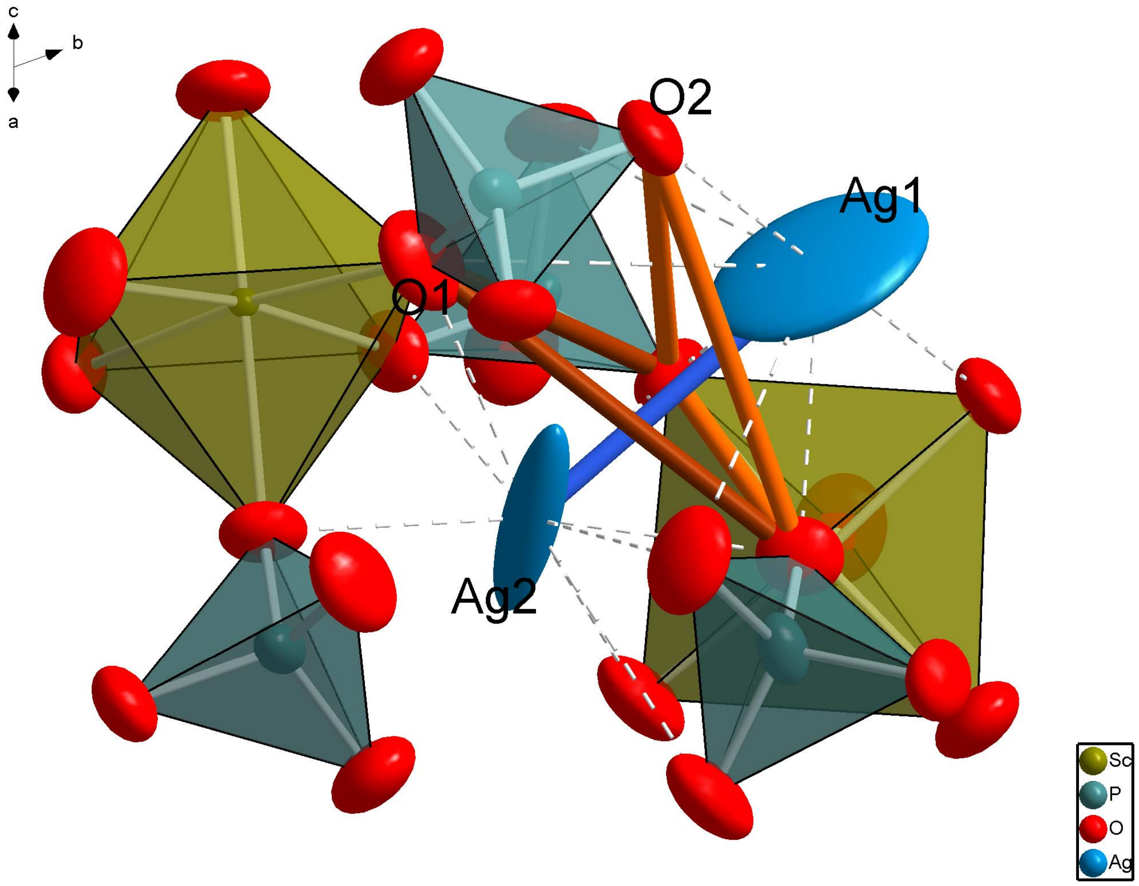

Figure S2 Part of the NaSICON structure of $Ag_3Sc_2(PO_4)_3$ at 298 K ($R\bar{3}c$) showing the bottleneck triangles defined by the O1 and O2 oxygen atoms for Na^+ passageways, viewed along $[2\bar{1}4]$.

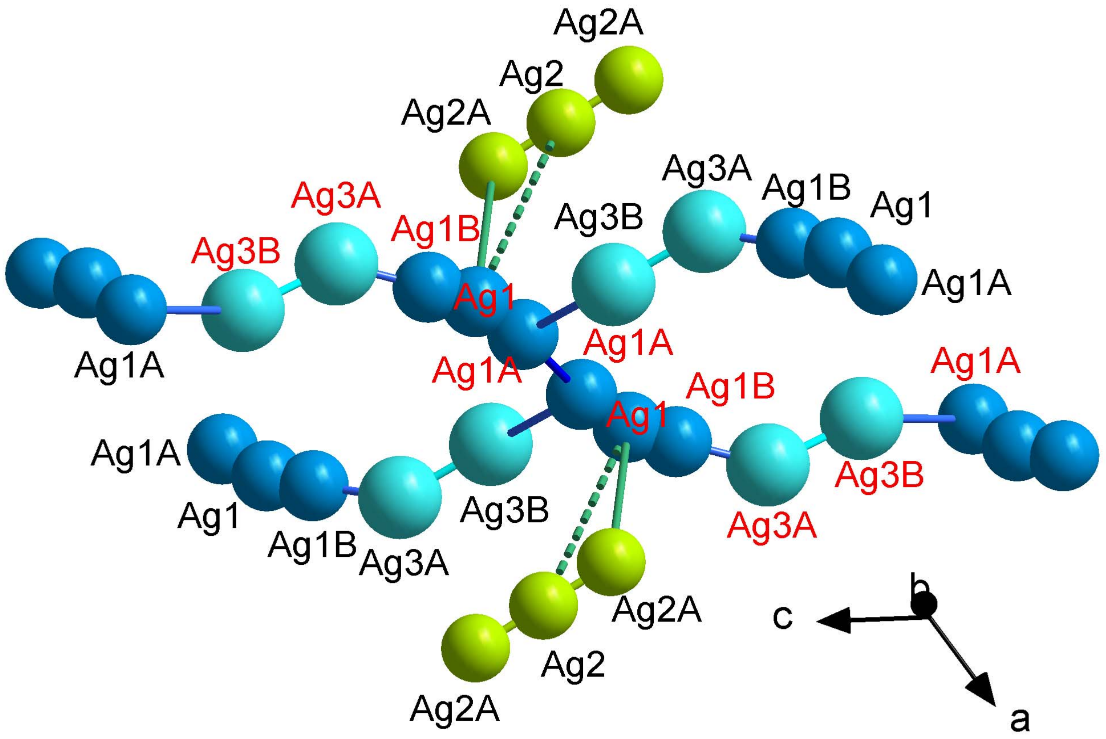

Figure S3 Possible diffusion pathway for Ag^+-Ag^+ migration in the low temperature α -phase of $Ag_3Sc_2(PO_4)_3$; shortest distances between Ag^+ - ions as described in the text are marked in red.

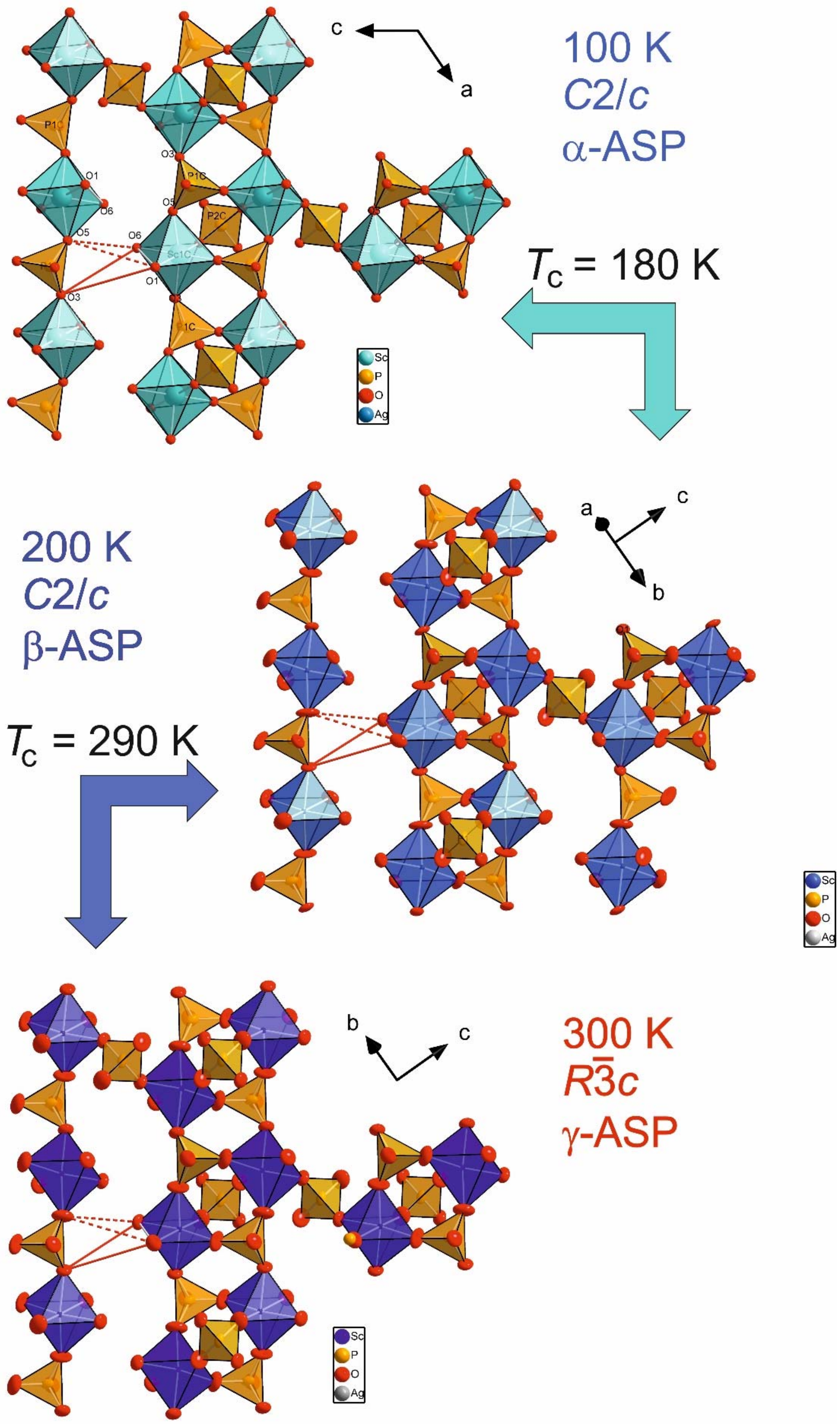

Figure S4 Comparison between the framework – structure of ASP in the three different modifications (a) *R*-3*c*, (b) *C*2/*c* β - phase viewed towards the (3 1 0) plane and (c) *C*2/*c* α - phase.
